# Supplementary material for: Efficient recovery and recycling/upcycling of precious metals using hydrazide-functionalized star-shaped polymers
Source: Nat Commun. 2024 May 8;15:3889. doi: 10.1038/s41467-024-48090-x (PMC11079046; doi:10.1038/s41467-024-48090-x)
Supplement: Supplementary file 1 — Supplementary Information [file 41467_2024_48090_MOESM1_ESM.pdf]

Efficient Recovery and Recycling/Upcycling of Precious Metals Using Hydrazide-Functionalized Star-Shaped Polymers

Seung Su Shin[1,4], Youngkyun Jung[2,4], Sungkwon Jeon[1], Sung-Joon Park[1], Su-Jin Yoon[2], Kyung-Won Jung[2], Jae-Woo Choi[2,3,*], Jung-Hyun Lee[1,*]

[1]Department of Chemical and Biological Engineering, Korea University, Seoul 02841, Republic of Korea.

[2]Center for Water Cycle Research, Korea Institute of Science and Technology, Seoul 02792, Republic of Korea.

[3]Division of Energy & Environment Technology, KIST School, Korea National University of Science and Technology, Seoul 02792, Republic of Korea.

[4]These authors contributed equally: Seung Su Shin and Youngkyun Jung.

E-mail: leejhyyy@korea.ac.kr; plead36@kist.re.kr

This file includes:

Supplementary Methods
Supplementary Notes
Supplementary Figures 1–37
Supplementary Tables 1–12
Supplementary References

Supplementary Methods

Polymer synthesis

S-PAcH(s) and S-PAcH(L) were synthesized by growing multiple PMAc linear arms on a CDx core through atom transfer radical polymerization (ATRP), followed by hydrazine functionalization. In detail, CDx (5 g) was dissolved in 1-methyl-2-pyrrolidone (NMP, 40.7 mL) under an Ar environment and then mixed with bromoisobutyryl bromide (BiBr, 34.3 g). The reaction took place at 25 °C for 24 h. After the reaction, impurities were removed by extraction with dichloromethane (DCM) and a saturated sodium carbonate (Na_2CO_3) aqueous solution. The organic phase solution was evaporated and vacuum-dried at room temperature to yield the BiBr-modified core (Br-CDx). To grow multiple PMAc linear arms via ATRP, Br-CDx (0.2 g), copper bromide (CuBr, 0.133 g), and *N*,*N*,*N*′,*N*″,*N*″-pentamethyldiethylenetriamine (PMDTA, 0.388 mL) were mixed with methyl acrylate (MAc, 24.82 mL for S-PMAc(s) and 74.46 mL for S-PMAc(L)) under an Ar atmosphere. After reacting at 75 °C for 48 h, the mixture was filtered through a silica/alumina-filled column to remove Cu residues. The filtered mixture was precipitated with methanol and then vacuum-dried at room temperature for 48 h to yield S-PMAc. For hydrazine functionalization, S-PMAc (5 g) and tetra-*n*-butyl ammonium bromide (TBABr, 5 g) were dissolved in tetrahydrofuran (THF, 150 mL), mixed with hydrazine hydrate (38 g), and reacted at 60 °C for 12 h. The mixture was cooled to 25 °C, and the aqueous phase containing S-PAcH was separated. S-PAcH was precipitated with methanol and purified via Soxhlet extraction with methanol at 80 °C for 48 h.

The linear counterpart of S-PAcH(s) (L-PAcH) was also synthesized via ATRP using MAc as the monomer and ethyl α-bromoisobutyrate (EBB) as the initiator, followed by hydrazine functionalization. EBB (0.14 mL), CuBr (0.133 g), and PMDTA (0.388 mL) were mixed, after which MAc (505.26 mL) was introduced to the mixture under an Ar atmosphere. After reacting at 75 °C for 48 h, the mixture was filtered through a silica/alumina-filled column to remove Cu residues. The filtered mixture was precipitated with methanol and vacuum-dried at room temperature for 48 h to yield L-PMAc, which was

subsequently functionalized with hydrazine following the same protocol used for the hydrazine functionalization of S-PMAc. The obtained S-PAcH and L-PAcH were dissolved in DI water for storage.

Adsorption isotherm analysis

Polymer adsorbents (10 mg) were added to the PM solutions (50 mL) at different C_i and pH 2. The mixture solution was stirred at 200 rpm for 3 h and then filtered through a PSF membrane. The PM concentration of the permeate solution was analyzed using an ICP-OES. The data of q_e versus C_e were fitted to three adsorption isotherm models, namely, the Langmuir, Freundlich, and Redlich–Peterson models.[1] The Langmuir model assumes that each of the active sites can adsorb only a single adsorbate in a monolayer, as given by

$$q_e = \frac{q_m K_L C_e}{1 + K_L C_e} \qquad (1)$$

where q_m is the maximum adsorption capacity and K_L is a Langmuir constant.

The Freundlich equation is an empirical model for multilayer adsorption, as given by

$$q_e = K_F C_e^{1/n} \qquad (2)$$

where K_F is the Freundlich constant and n is a constant related to the adsorption capacity and favorability.

The Redlich–Peterson model is a hybrid of the Langmuir and Freundlich models and can be expressed as

$$q_e = \frac{K_R C_e}{1 + a_R C_e^{\alpha}} \qquad (3)$$

where K_R and a_R are the Redlich–Peterson constants and α is an exponent ranging from 0 to 1. The above equation is reduced to the Freundlich model when $a_R C_e^{\alpha} >> 1$ and the Langmuir model when $\alpha = 1$.

Adsorption kinetics analysis

S-PAcH (10 mg) was added to the PM (200 mg L^{-1}) solutions (50 mL) at pH 2 and stirred at 200 rpm for 1–180 min. After a predetermined contact time (t), the mixture was filtered through a PSF membrane, and the PM concentration of the permeate solution was analyzed using an ICP-OES. The data of PM

adsorption capacity (q_t) versus t were fitted to two kinetics models, namely, the pseudo-first-order and pseudo-second-order models.[2] The pseudo-first-order model assumes that adsorption occurs via physisorption on a few adsorptive sites and that the adsorption rate is predominantly affected by the diffusion rate of the adsorbate, as described by

$$q_t = q_e\left(1 - e^{-k_1 t}\right) \quad (4)$$

where k_1 is the rate constant.

The pseudo-second-order model assumes that adsorption occurs via chemisorption on abundant adsorptive sites, as given by

$$q_t = \frac{k_2 q_e^2 t}{1 + k_2 q_e t} \quad (5)$$

where k_2 is the rate constant.

Note that reliable kinetics analyses of bPEI and PAAm could not be performed because these adsorbents could not be collected rapidly using membrane filtration.

PM recovery of S-PAcH(L) under extremely acidic conditions

PM (50 mg L^{-1}) solutions were prepared by diluting the respective PM (1000 mg L^{-1}) standard solutions with DI water while adjusting their HCl concentrations to 1−5 M using a HCl (37%) aqueous solution. S-PAcH(L) (10 mg) was added to the PM solutions (50 mL) and stirred at 200 rpm for 3 h. The mixture was centrifuged at 9400 g for 10 min, and the supernatant (0.5 mL) was diluted 100-fold using DI water. The diluted solution was then filtered through a PSF ultrafiltration membrane, and the supernatant was collected. The PM ion concentrations of the solutions obtained before and after the addition of S-PAcH(L) were measured using an ICP-OES to quantify the R_e.

Effect of organic pollutants on PM recovery by S-PAcH(L)

NOM, FA, and HA were selected as organic pollutants which are present in industrial water[3]. PCBs, PBDEs, PAHs, and PAEs were also selected as organic pollutants which can be generated during the

leaching process of e-waste[4]. PM (200 mg L^{-1}) aqueous solutions at pH 2 were mixed with each organic pollutant (200 mg L^{-1}) or all combined pollutants (200 mg L^{-1} each). S-PAcH(L) (10 mg) was added to the organic pollutant-containing PM solutions (50 mL) and stirred at 200 rpm for 3 h. The mixture was then filtered through a PSF ultrafiltration membrane, and the supernatant was collected. The PM ion concentrations of the solutions obtained before and after the addition of S-PAcH(L) were measured using an ICP-OES to quantify the R_e.

Turnover number (TON) and turnover frequency (TOF) analysis

To estimate the catalytic activity of PM/S-PAcH(L) for dye reduction, its TON and TOF values were determined as follows. S-PAcH(L) (10 mg) was added to the PM (200 mg L^{-1}) solutions (50 mL) at pH 2 and then stirred at 200 rpm for 30 min. The precipitates formed in the solution were collected by PSF membrane filtration, vacuum-dried at room temperature, and then redispersed in DI water to obtain PM/S-PAcH(L)-containing solutions at predetermined PM/S-PAcH(L) concentrations. Each of these solutions (1.5 mL) was added to a dye (4-NP or MO, 0.02 mM)/$NaBH_4$ (2 mM) aqueous solution (1.5 mL). Dye reduction was then monitored as a function of time using an UV-vis spectrophotometer. TON represents the consumed amount of a reactant per unit amount (mass or mole) of a catalyst. TON denotes the consumed amount of a reactant per unit amount of a catalyst per unit time. The TON and TOF values of PM/S-PAcH(L), which acts as a heterogeneous catalyst for dye reduction, can be practically calculated, as given by[5–7]

$$\mathrm{TON} = C_{\mathrm{dye}}/C_{\mathrm{cat}} \qquad (6)$$

where C_{cat} denotes the lowest concentration of the catalyst (PM/S-PAcH(L)) that leads to complete dye reduction at a given dye concentration (C_{dye}).

$$\mathrm{TOF} = \mathrm{TON}/t \qquad (7)$$

where t denotes the shortest reaction time to reach complete dye reduction.

At the given dye (4-MP and MO) concentrations (C_{dye} = 0.02 mM), C_{cat} and t of PM/S-PAcH(L) were determined from Supplementary Fig. 32, and its TON and TOF values were calculated and compared with those of other reported catalysts, as summarized in Supplementary Tables 9 and 10.

Supplementary Notes

Supplementary Note 1. Oxidation-reduction (redox) reaction mechanisms between S-PAcH and PM ions

The redox reaction, oxidation half-reaction, and reduction half-reaction between the hydrazide $-NH_2$ of S-PAcH and Au ions ($AuCl_4^-$) can be described as follows[8].

(redox reaction) $-NH_2 + 2AuCl_4^- + 2H_2O \rightarrow 2Au + -NO_2 + 6H^+ + 8Cl^-$ (Supplementary Eq. 1)

(oxidation half-reaction) $-NH_2 + 2H_2O \rightarrow -NO_2 + 6H^+ + 6e^-$ (Supplementary Eq. 2)

(reduction half-reaction) $AuCl_4^- + 3e^- \rightarrow Au + 4Cl^-$ (Supplementary Eq. 3)

Based on the above redox reaction mechanism, the reaction between $-NH_2$ of S-PAcH and Pd ions ($PdCl_4^{2-}$) can be depicted as follows.

(redox reaction) $-NH_2 + 3PdCl_6^{2-} + 2H_2O \rightarrow 3Pd + -NO_2 + 6H^+ + 12Cl^-$ (Supplementary Eq. 4)

(oxidation half-reaction) $-NH_2 + 2H_2O \rightarrow -NO_2 + 6H^+ + 6e^-$ (Supplementary Eq. 5)

(reduction half-reaction) $PdCl_4^{2-} + 2e^- \rightarrow Pd + 4Cl^-$ (Supplementary Eq. 6)

The reaction between $-NH_2$ of S-PAcH and Pt ions ($PtCl_6^{2-}$) can also be depicted as follows.

(redox reaction) $-2NH_2 + 3PtCl_4^- + 4H_2O \rightarrow 3Pt + -NO_2 + 12H^+ + 18Cl^-$ (Supplementary Eq. 7)

(oxidation half-reaction) $-NH_2 + 2H_2O \rightarrow -NO_2 + 6H^+ + 6e^-$ (Supplementary Eq. 8)

(reduction half-reaction) $PtCl_6^{2-} + 4e^- \rightarrow Pt + 6Cl^-$ (Supplementary Eq. 9)

Based on the above reaction mechanisms, it is evident that one $-NH_2$ group of S-PAcH provides six electrons during its oxidation to $-NO_2$, thus reducing multiple PM ions (i.e., 2 for $AuCl_4^-$, 3 for $PdCl_4^{2-}$, and 1.5 for $PtCl_6^{2-}$) to PM metals.

Supplementary Note 2. Determination of the optimal pH condition for PM adsorption tests

The optimal pH for the PM adsorption tests was identified by characterizing the R_e of the adsorbents (i.e., S-PAcH(L) and commercial amine polymers) as a function of pH (Supplementary Figs. 19–21). All adsorbents exhibited higher R_e values at lower pH values, particularly for Au. This can be attributed to the enhanced positive charges on the absorbents, resulting in intensified electrostatic attraction with anionic PM species[9]. Likewise, the lower R_e of S-PAcH(L) under basic conditions (pH >8) can be attributed to reinforced electrostatic repulsion between S-PAcH(L) and anionic PM species[10]. The Pd adsorption tests were valid only at pH <5 because Pd ions are precipitated at pH ≥5[11]. S-PAcH(L) maintained its very high R_e (>99%) for all PMs even at 1M HCl (corresponding to ~0 pH) (Supplementary Fig. 23). However, its R_e value for all PMs slightly decreased at 3M HCl and drastically decreased at 5M HCl. We attributed the reduced R_e of S-PAcH(L) under extremely acidic conditions (>1M HCl) presumably to the hydrolysis of the hydrazide groups of S-PAcH[12].

Supplementary Note 3. Comparison of the PM reduction capabilities of S-PAcH and reducing agents

The PM reduction capabilities of S-PAcH(L) and reducing agents (hydrazine and $NaBH_4$) were characterized and compared by examining the PM (200 mg L^{-1}) solutions (50 mL) at pH 2, to which the same dose (0.2 g L^{-1}) of S-PAcH(L), hydrazine, and $NaBH_4$ was added. Like S-PAcH(L), both reducing agents reduced all PM ions to PM NPs, followed by precipitation, except for hydrazine in the Pt-ion-containing solution, in which no Pt reduction was observed (Supplementary Fig. 25). This result indicates that hydrazine has a significantly lower Pt reduction capability than S-PAcH(L) and $NaBH_4$, which was also supported by the previous finding that highly concentrated hydrazine is required for reducing Pt ions[13] Consistently, we observed that Pt ions were reduced to Pt NPs at a very high hydrazine concentration (150 g L^{-1}), as shown in Supplementary Fig. 26.

Supplementary Note 4. Comparison of the PM recovery efficiency and selectivity of S-PAcH and reducing agents

Based on the experiments shown in Supplementary Fig. 25, the R_e values of S-PAcH(L) and reducing agents were estimated and compared, as shown in Supplementary Fig. 27. Both reducing agents exhibited remarkably lower R_e for all PMs compared with S-PAcH(L). The significantly low Pt reduction capability of hydrazine can account for its marginal R_e for Pt (~0%) under the given conditions. The higher R_e of S-PAcH(L) than those of the reducing agents can be attributed to its high reduction capability combined with its adsorption mechanism via electrostatic and chelation interactions. This result highlights the beneficial feature of PAcH(L) with both adsorbent and reductant functions, which synergistically improve PM adsorption performance above that achievable by reducing agents with a reduction function only.

The PM selectivity of the reducing agents was also characterized and compared with that of S-PAcH(L) (Supplementary Fig. 29). As mentioned in the main text, for all the simulated (CPU and spent catalyst leachates and groundwater) feed solutions, S-PAcH(L) exhibited ~100% R_e for all PMs but negligible R_e for other co-existing metal cations, indicating its excellent selectivity toward all PMs. In contrast, both reducing agents displayed significantly lower selectivity toward PMs than S-PAcH(L) as follows.

(1) Simulated CPU leachate: Compared with S-PAcH(L), both reducing agents exhibited lower R_e for Au and remarkably higher R_e for Cu (31.4 % for hydrazine and 26.4% for $NaBH_4$) (Supplementary Fig. 29a), indicating their lower selectivity toward Au. Together with Au ions, Cu ions with high reduction potential (0.34 V) were likely to be reduced to Cu NPs and precipitated by reducing agents[14].

(2) Simulated spent catalyst leachates: Both reducing agents exhibited significantly lower R_e for Pd and Pt (even negligible R_e for Pt in the case of hydrazine) than S-PAcH(L) (Supplementary Figs. 29b and c). Interestingly, despite the low reduction potential of Al (−1.66 V), $NaBH_4$ exhibited significantly higher R_e for Al (41.5% and 19.8% for the simulated Pd and Pt spent catalyst leachates, respectively) than S-PAcH(L). Because borohydride ions (BH_4^-) can act as a stabilizer for metal NPs[15,16], cationic Al ions were likely to be adsorbed onto anionic BH_4^- ion-bound Pd or Pt NPs via electrostatic attraction, and thus,

recovered with PM NPs. Therefore, for the simulated spent catalyst leachates, both reducing agents exhibited significantly lower selectivity toward Pd and Pt compared with S-PAcH(L).

(3) Simulated groundwater: Both reducing agents selectively recovered PMs by preferentially reducing PM ions with significantly higher reduction potentials than those of co-existing metal ions, except for Pt recovery by hydrazine whose Pt reduction capability is low. As a result, for the simulated groundwater, both reducing agents exhibited high selectivity toward PMs, comparable to that of S-PAcH(L), except for hydrazine whose selectivity toward Pt was marginal owing to its low Pt reduction capability.

These experimental results clearly demonstrate the superior PM selectivity of S-PAcH(L), which can be attributed to its strong reduction capability combined with electrostatic repulsion between positively charged S-PAcH(L) and co-existing metal cations, as mentioned in the main text. This result further emphasizes the beneficial feature of S-PAcH, whose combined adsorbent and reductant functions enables selective PM recovery.

Supplementary Note 5. Comparison of the cost effectiveness of S-PAcH and reducing agents for PM recovery

It is very difficult to accurately estimate the price of S-PAcH(L), which is a lab-scale sample, because the accurate information of additional production costs (e.g., facilities, plant planning, labor, etc.) and the industrial-grade prices of raw materials is not available. Nevertheless, we can roughly evaluate the final price of S-PAcH(L) based on the reagent-grade prices of the raw materials required for its lab-scale synthesis. The pure price of S-PAcH(L) calculated based on the amounts and unit prices of the raw materials needed for synthesizing S-PAcH(L) of 1 kg was ~\$6961.3 kg^{-1}, as summarized in Supplementary Table 7. With the assumption that raw material costs occupy approximately 60–90% of the total production cost[17], the final price of S-PAcH(L) can be reasonably estimated to be \$7734.8–11602.2 kg^{-1}. However, this calculation is a very rough estimation, and the accurate total price should be estimated by manufacturers.

To fairly assess the cost effectiveness of S-PAcH(L) and reducing agents, their costs required for recovering the same amount of PM were compared. To do this, we first identified the doses of the reducing agents that could achieve the same R_e as that achieved by the dose (0.2 g L^{-1}) of S-PAcH(L) at a given PM ion concentration (200 mg L^{-1}), as shown in Supplementary Fig. 30. Although both reducing agents exhibited lower R_e for all PMs compared with S-PAcH(L) at the same dose (0.2 g L^{-1}), their R_e values increased with their doses, except for hydrazine whose R_e for Pt remained marginal (~0%) even at the highest dose investigated (5.0 g L^{-1}) owing to its very low Pt reduction capability.

Based on the prices of S-PAcH(L) and reducing agents and their doses required for achieving the same R_e at a given PM ion concentration (200 mg L^{-1}) (determined from Supplementary Fig. 30), their costs for PM recovery were calculated, as summarized in Supplementary Table 8. The material price of S-PAcH(L) was notably higher than those of hydrazine and $NaBH_4$. Nevertheless, the cost of S-PAcH(L) for PM recovery was similar or even lower compared with hydrazine and slightly higher compared with $NaBH_4$ owing to the superior PM adsorption capacity of S-PAcH(L). We also need to consider the PM selectivity and recoverability of adsorbents and reducing agents to assess their cost effectiveness for practical PM recovery. The significantly lower PM selectivity of reducing agents than that of S-PAcH(L) would incur additional costs for PM separation. Furthermore, the small molecular size of reducing agents (unreacted and/or reacted forms) renders their recovery/collection difficult[18], which requires additional costs for purification/recovery processes. Particularly, in a PM reduction process using $NaBH_4$, toxic impurities, such as boric acid and sodium metaborate, are generated, which should be removed by employing additional separation processes[18]. Based on these considerations, we believe that our S-PAcH polymers with higher PM adsorption capacity/selectivity and recoverability would be more cost-effective compared with reducing agents.

Supplementary Note 6. Effect of organic pollutants on PM recovery by S-PAcH(L)

We identified the effect of coexisting organic pollutants on PM recovery by S-PAcH(L) by evaluating the R_e of S-PAcH(L) using PM solutions containing various organic pollutants (natural organic matter [NOM], fulvic acid [FA], humic acid [HA], polychlorinated biphenyls [PCBs], polybrominated diphenyl ethers [PBDEs], polyaromatic hydrocarbons [PAHs], and phthalate esters [PAEs]), as shown in Supplementary Fig. 31. The R_e values of S-PAcH(L) for all PMs were similar for the PM solutions with and without (control) organic pollutants. This result clearly demonstrates that coexisting organic pollutants cannot impair the PM reduction capability, and thus, recovery performance of S-PAcH(L). This could be attributed to 1) poor affinity between highly hydrophilic S-PAcH and relatively hydrophobic organic pollutants (i.e., low hydrophobic interactions)[4] and 2) rapid PM adsorption by S-PAcH(L), which could significantly reduce the probability of organic pollutants to form complexes with S-PAcH(L).

Supplementary Note 7. Assessment of the cost effectiveness of S-PAcH(L) depending on the PM concentration

We calculated the price (value) ratio of adsorbed PM to S-PAcH(L) in PM/S-PAcH(L)s formed with different PM ion concentrations based on the weight and price of adsorbed PM and S-PAcH(L), as summarized in Supplementary Tables 7 and 12. Two PM ion concentrations, (1) 200 mg L^{-1} and (2) PM concentrations in the leachates of real-world CPU and spent Pd and Pt catalyst samples, were considered. As shown in Supplementary Table 12, at the PM concentration of 200 mg L^{-1}, the price of adsorbed (recovered) PM was 1.9 to 8.5 times higher than that of S-PAcH(L). Although more extensive cost analysis is needed, in this case where the value of recovered PM exceeds that of S-PAcH(L) owing to high PM adsorption capacity, simple calcination for PM recovery could be more cost-effective than a relatively complex regeneration process[19,20]. Conversely, in the case of real-world leachates with low PM concentrations (~10 mg L^{-1}), the price of S-PAcH(L) was higher than that of adsorbed PM. In this case, the regeneration (reuse) of S-PAcH(L) could be more cost-effective than calcination which results in the loss of S-PAcH(L).

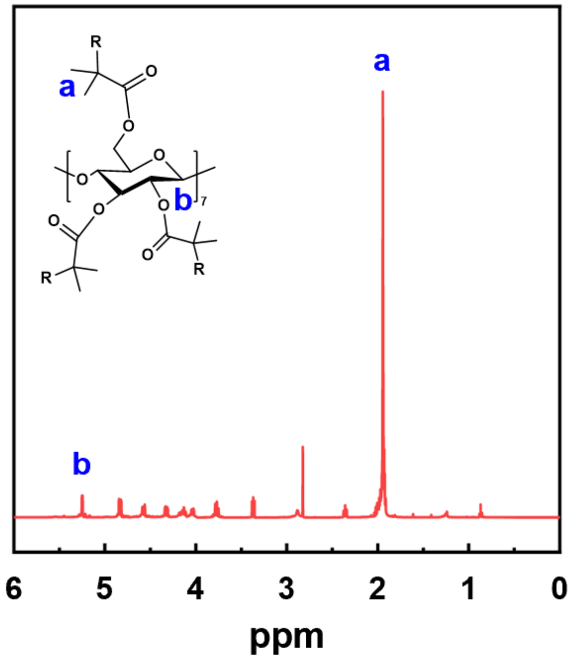

Supplementary Fig. 1. ^{1}H NMR spectrum of Br-CDx. The conversion (E) of the hydroxyl groups on a CDx to BiBr was calculated from the ^{1}H NMR spectrum of Br-CDx using the following equation: E (%) = 100 × $A_a/18A_b$, where A_a and A_b are the integral areas of the peaks corresponding to a and b protons, respectively[21]. Based on this calculation, E was determined to be ~90%, indicating that ~19 BiBr initiators were attached to a single CDx core. Because each BiBr initiator equally grows polymer arms by initiating the ATRP reaction, the arm number of the synthesized S-PAcH polymer can be reasonably assumed to be ~19.

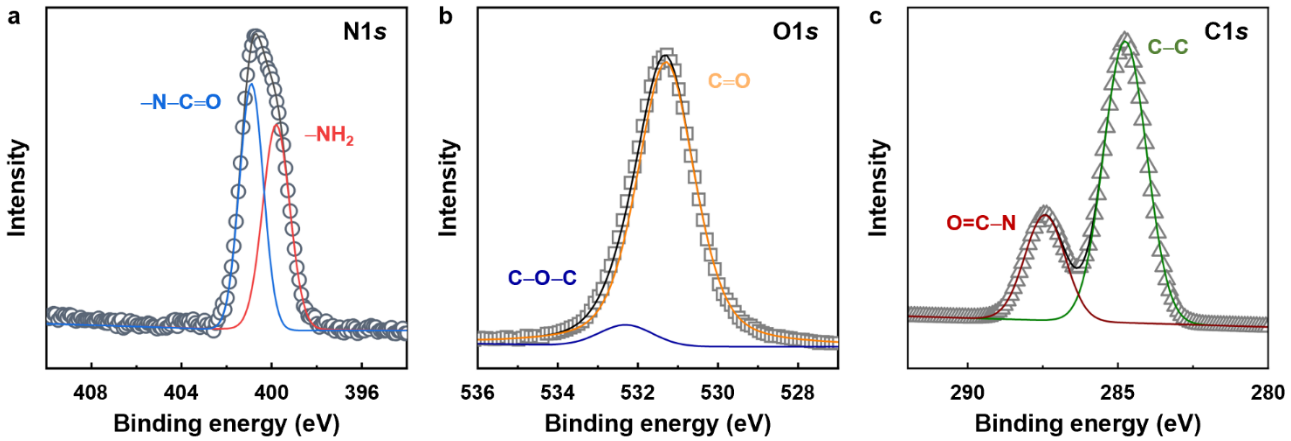

Supplementary Fig. 2. Deconvolution of the high-resolution (**a**) N1*s*, (**b**) O1*s*, and (**c**) C1*s* XPS peaks of S-PAcH.

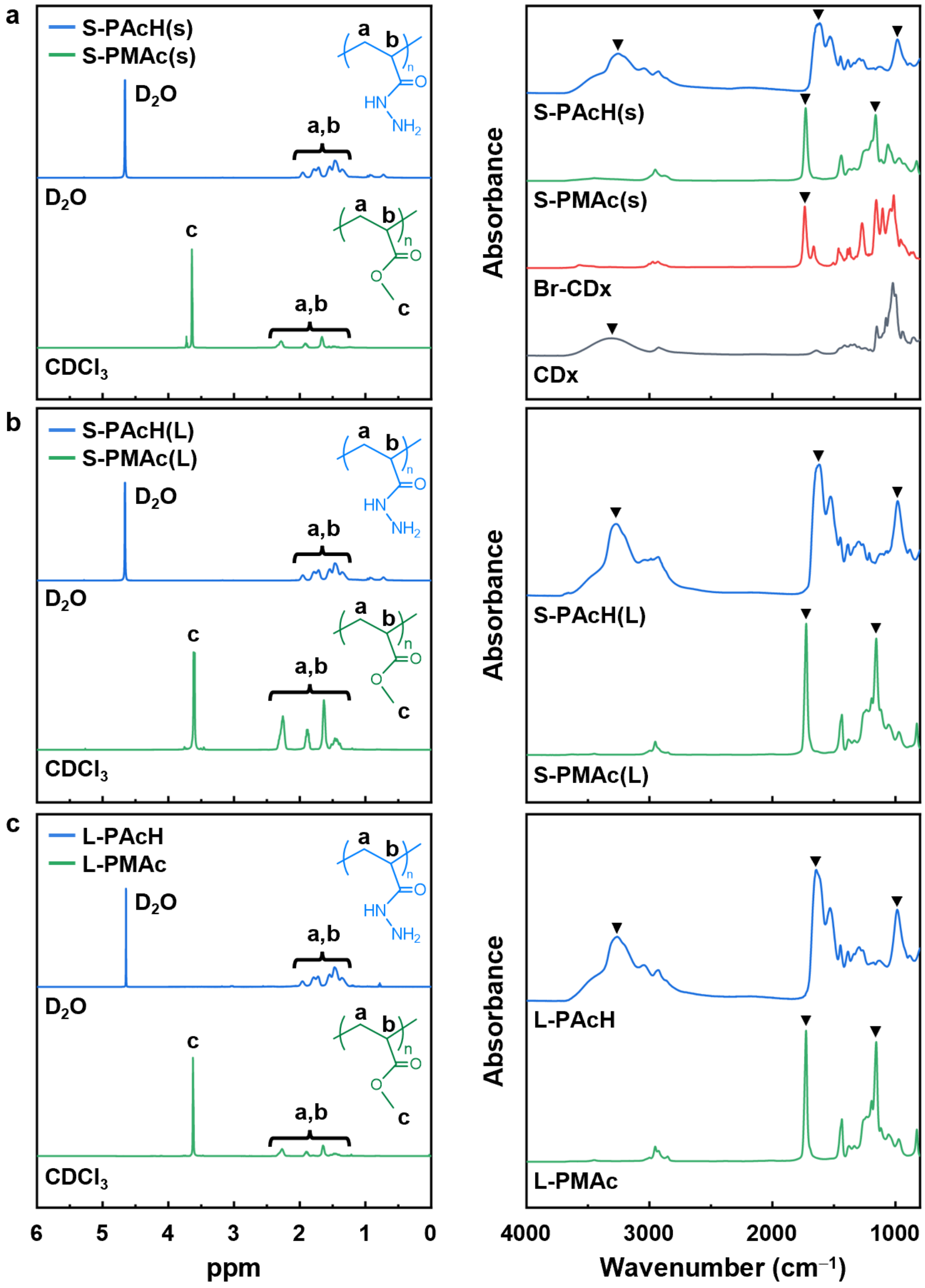

Supplementary Fig. 3. ^{1}H NMR (left) and FT-IR (right) spectra of the compounds obtained at each step of the synthesis of (**a**) S-PAcH(s), (**b**) S-PAcH(L), and (**c**) L-PAcH. The attachment of BiBr initiators onto CDx was confirmed by the FT-IR spectrum of Br-CDx, where the ester group peak at 1728 cm^{-1} (ester C=O stretching) appeared, whereas the hydroxyl group peak of CDx at 3350 cm^{-1} (O–H stretching) disappeared[22]. Polymer growth via ATRP was confirmed by the appearance of the ester group peaks of PMAc at 1728 cm^{-1} (ester C=O stretching) and 1158 cm^{-1} (ester C–O stretching) in the FT-IR spectra of the PMAc-series polymers[22]. After hydrazine functionalization, FT-IR analysis revealed PAcH peaks at 3300 cm^{-1} (amide N–H stretching), 1610 cm^{-1} (amide C=O stretching), and 981 cm^{-1} (amide C–N stretching), thus confirming complete hydrazine functionalization[22].

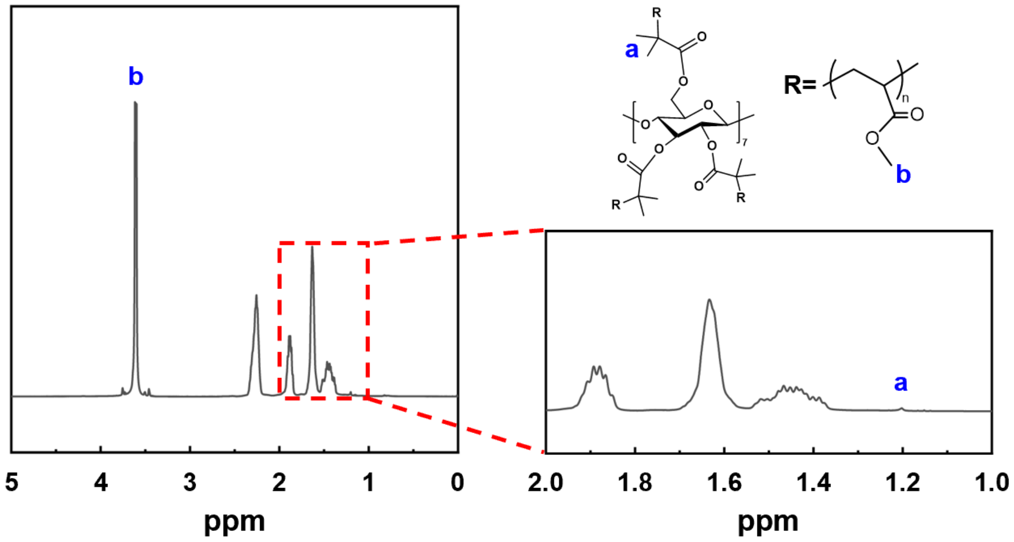

Supplementary Fig. 4. ^{1}H NMR spectra of S-PMAc. The total number of the PMAc repeating units (N_{PMAc}) of S-PMAc was calculated as follows: $N_{PMAc} = 38A_b/A_a$, where A_a and A_b are the integral areas of the peaks corresponding to a and b protons, respectively[22]. The total M_w of the PMAc arms ($M_{w,PMAc}$) of S-PAcH was calculated by multiplying N_{PMAc} by the M_w (= 86.09 g mol^{-1}) of the PMAc repeating unit as follows: $M_{w,PMAc} = N_{PMAc} \times 86.09$. Because the M_w values of the PMAc and PAcH repeating units are identical, the total M_w of the PAcH arms ($M_{w,PAcH}$) of S-PAcH is equal to $M_{w,PMAc}$. The M_w of each PAcH arm ($M_{w,arm}$) of S-PAcH can be determined by dividing $M_{w,PAcH}$ by the total number of PAcH arms (19). The calculated $M_{w,arm}$ values of S-PAcH(s) and S-PAcH(L) were 7.37 and 16.53 kg mol^{-1}, respectively.

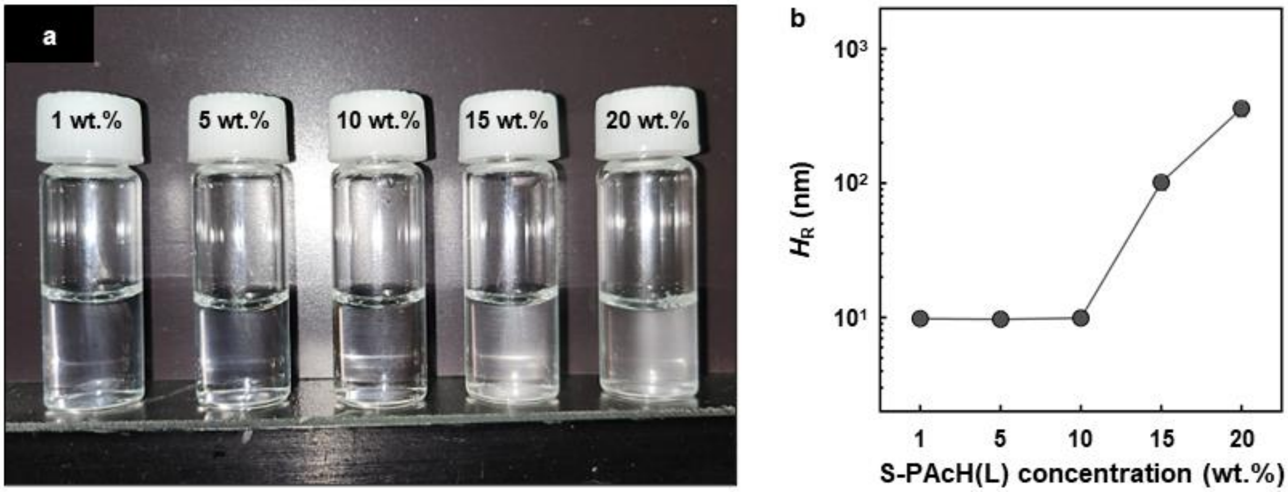

Supplementary Fig. 5. (**a**) Photographs and (**b**) hydrodynamic diameter (H_R) of the S-PAcH(L) aqueous solutions at different S-PAcH(L) concentrations. Error bars represent standard deviations determined from three replicates.

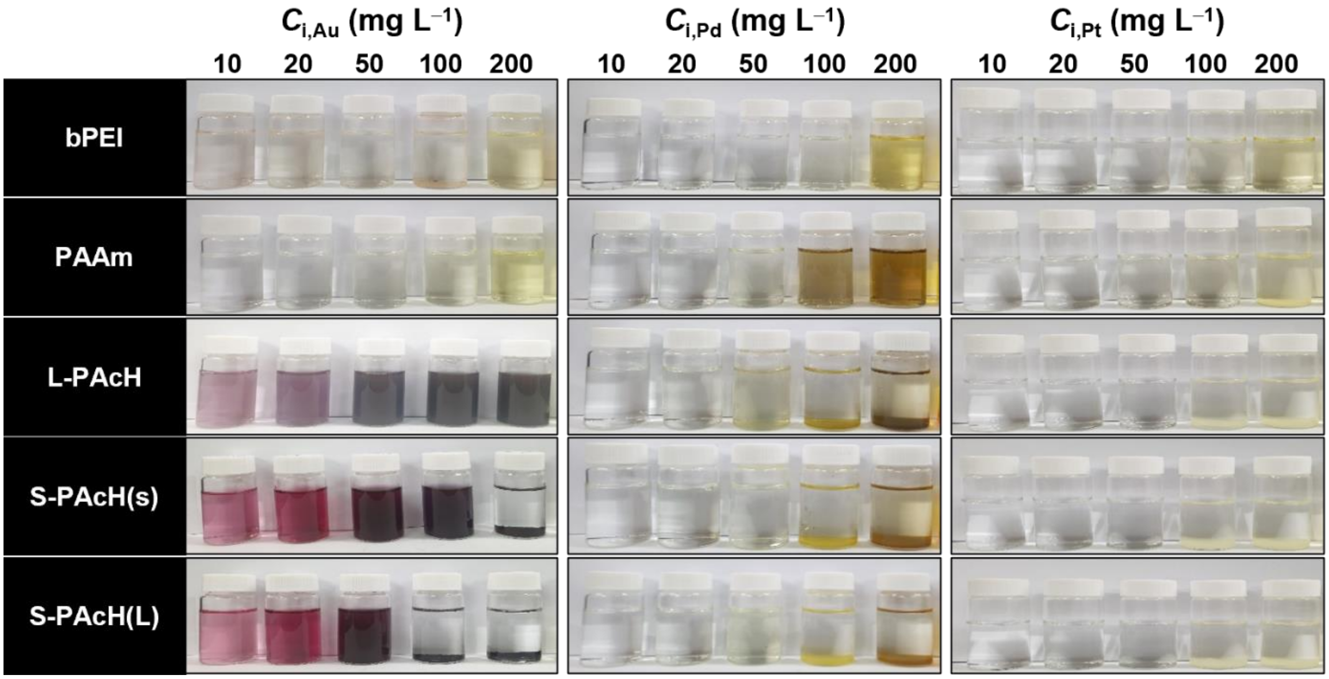

Supplementary Fig. 6. Photographs of the PM aqueous solutions at different initial PM ion concentrations ($C_{i,PM}$) after the addition of the polymer adsorbents (polymer concentration = 0.2 g L^{-1}, solution pH = 2, contact time = 3 h).

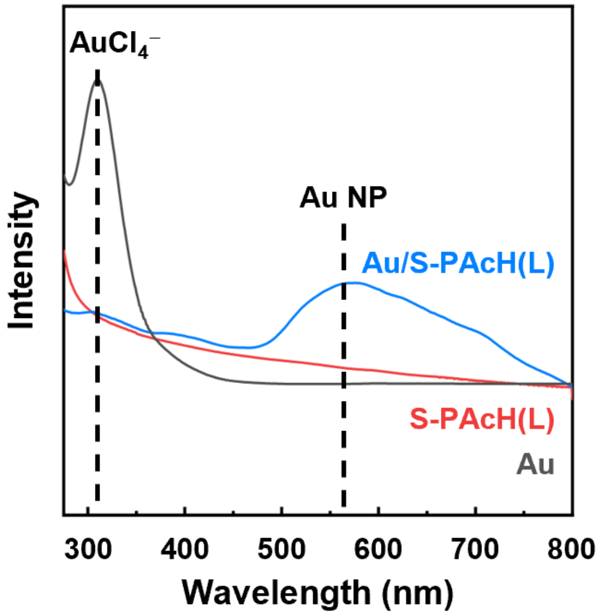

Supplementary Fig. 7. UV-vis spectra of Au ion ($AuCl_4^-$, 200 mg L^{-1}), S-PAcH(L), and Au/S-PAcH(L) aqueous solutions (S-PAcH(L) concentration = 0.2 g L^{-1}, solution pH = 2, contact time = 3 h).

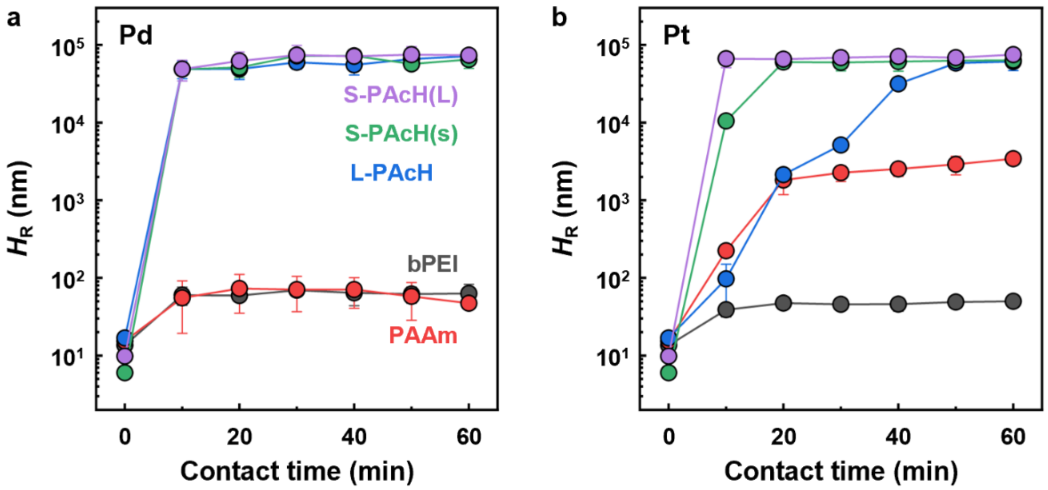

Supplementary Fig. 8. Hydrodynamic diameter (H_R) of the polymers after their addition (0.2 g L^{-1}) to (**a**) Pd and (**b**) Pt aqueous solutions (PM ion concentration = 200 mg L^{-1}, solution pH = 2) as a function of contact time. Error bars represent standard deviations determined from three replicates.

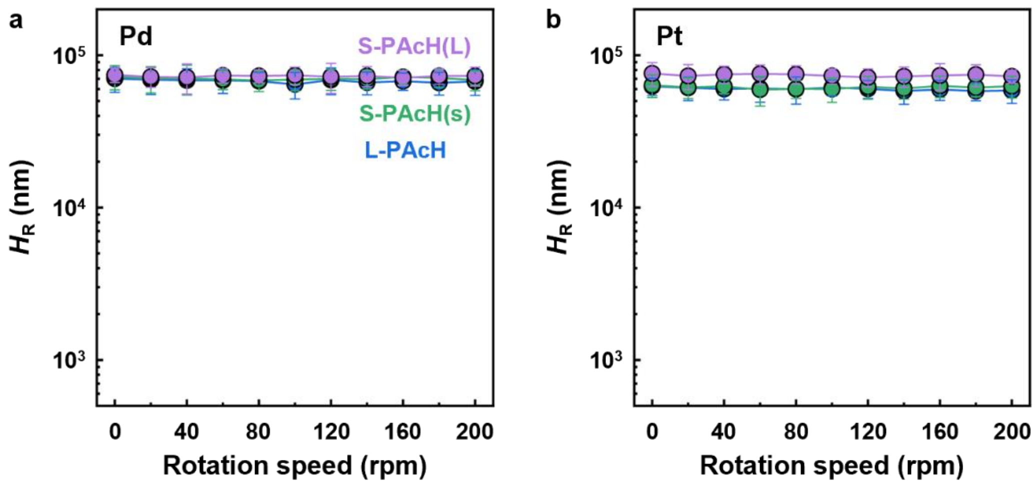

Supplementary Fig. 9. Hydrodynamic diameter (H_R) of the PM/PAcH precipitates as a function of rotation speed: (**a**) Pd and (**b**) Pt. The precipitates were formed by allowing contact between PAcH-series polymers (0.2 g L^{-1}) and PM (200 mg L^{-1}) aqueous solutions (pH = 2) for 24 h. Error bars represent standard deviations determined from three replicates.

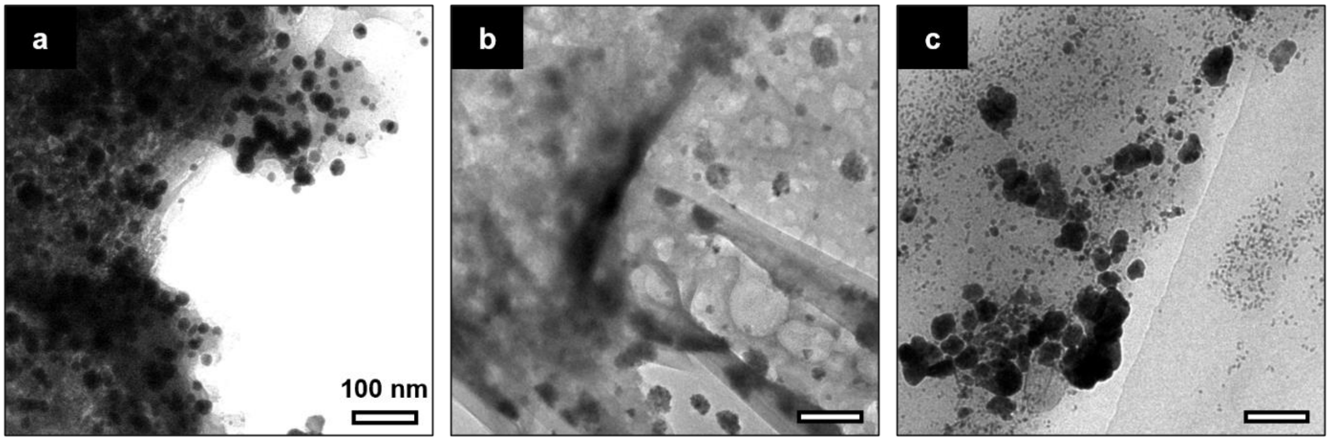

Supplementary Fig. 10. TEM images of the PM/S-PAcH(L) precipitates: (**a**) Au, (**b**) Pd, and (**c**) Pt. The precipitates were formed by allowing contact between S-PAcH(L) (0.2 g L^{-1}) and PM (200 mg L^{-1}) aqueous solutions (pH = 2) for 3 h.

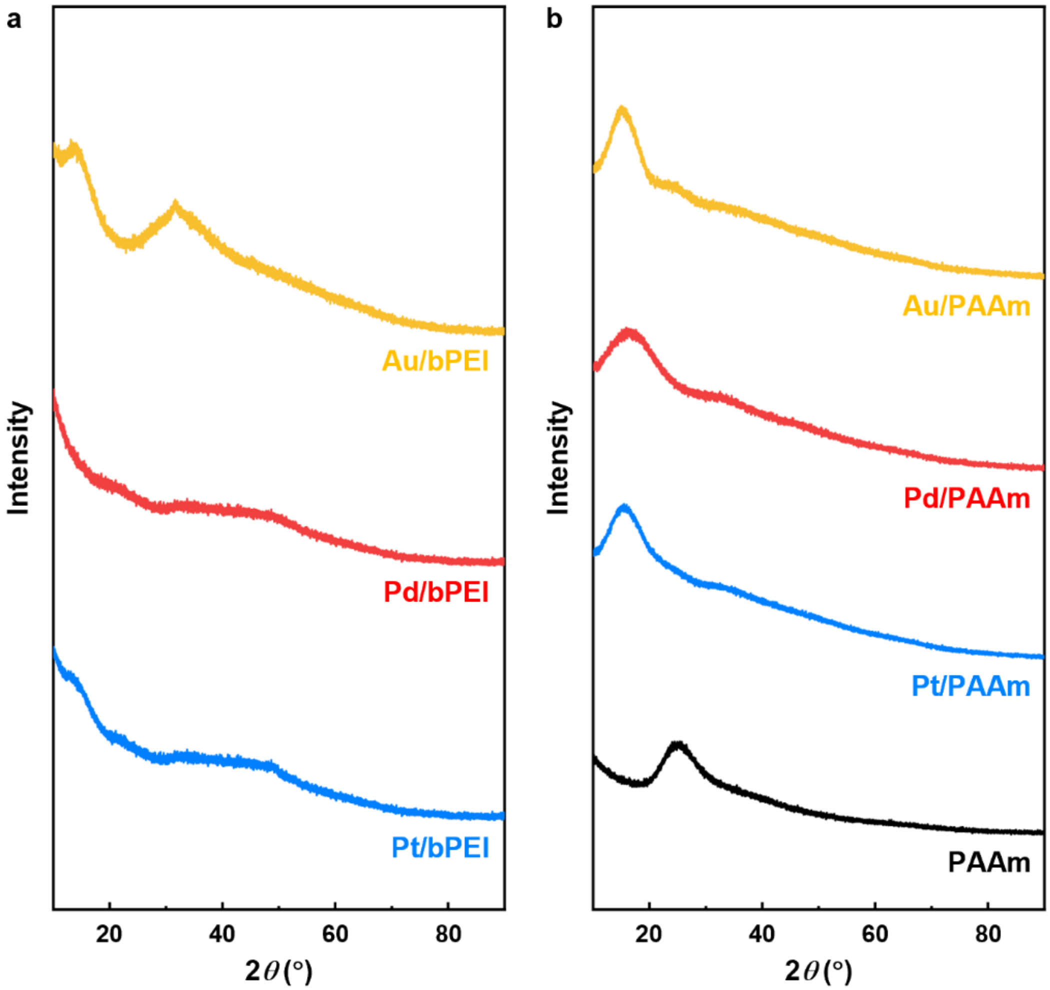

Supplementary Fig. 11. XRD patterns of the pristine commercial amine and PM/amine polymers: (**a**) bPEI and (**b**) PAAm. The PM/amine polymers were formed by allowing contact between amine polymers (0.2 g L^{-1}) and PM (200 mg L^{-1}) aqueous solutions (pH = 2) for 3 h. The XRD pattern of pristine bPEI could not be obtained because a powder-like sample could not be obtained even after vacuum-drying for 30 d.

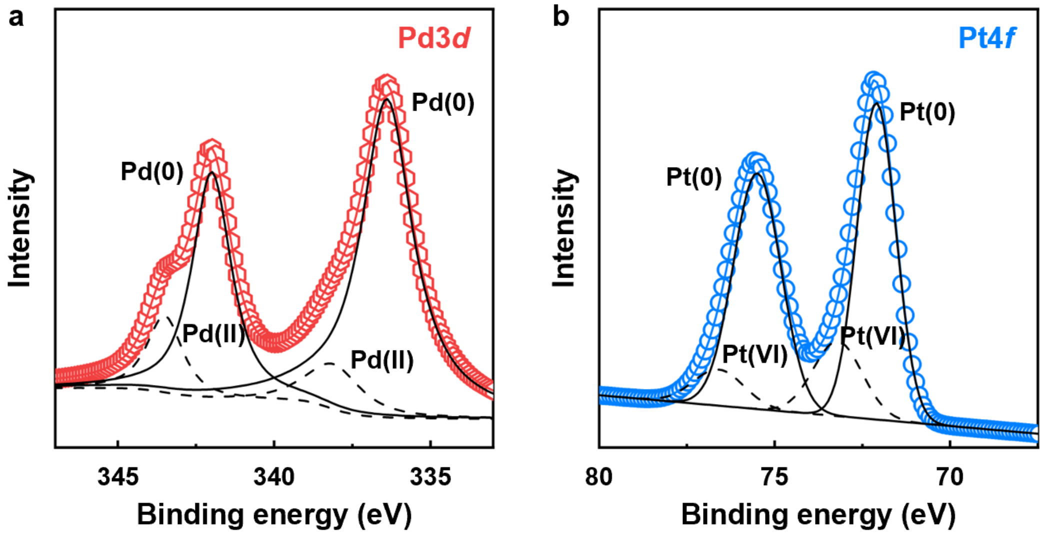

Supplementary Fig. 12. Deconvolution of the high-resolution (**a**) Pd3*d* and (**b**) Pt4*f* XPS peaks of the PM/S-PAcH(L) precipitates. The precipitates were formed by allowing contact between S-PAcH(L) (0.2 g L^{-1}) and PM (200 mg L^{-1}) aqueous solutions (pH = 2) for 3 h.

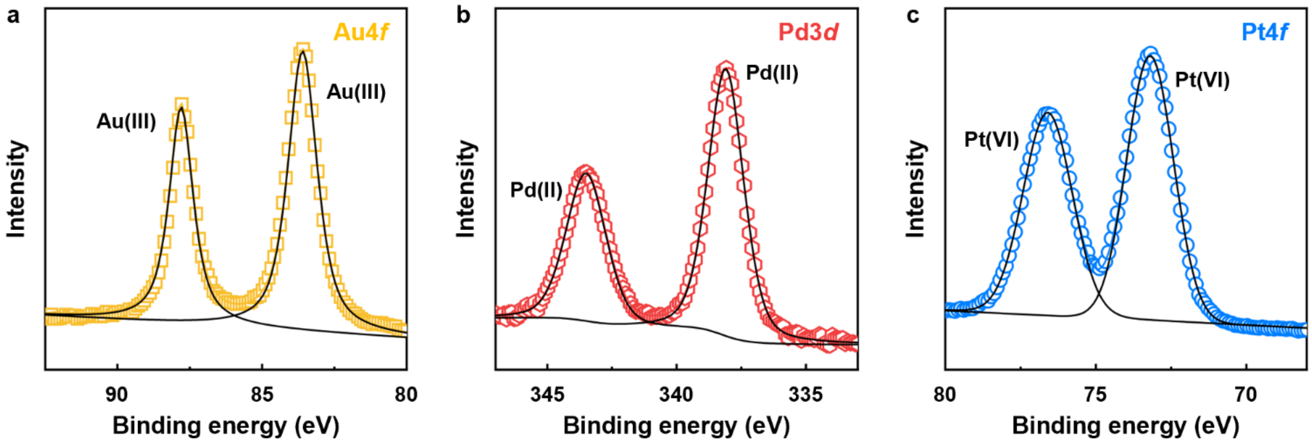

Supplementary Fig. 13. Deconvolution of the high-resolution (**a**) Au4*f*, (**b**) Pd3*d*, and (**c**) Pt4*f* XPS peaks of the PM/bPEI polymers. The PM/bPEI polymers were formed by allowing contact between bPEI (0.2 g L^{-1}) and PM (200 mg L^{-1}) aqueous solutions (pH = 2) for 3 h.

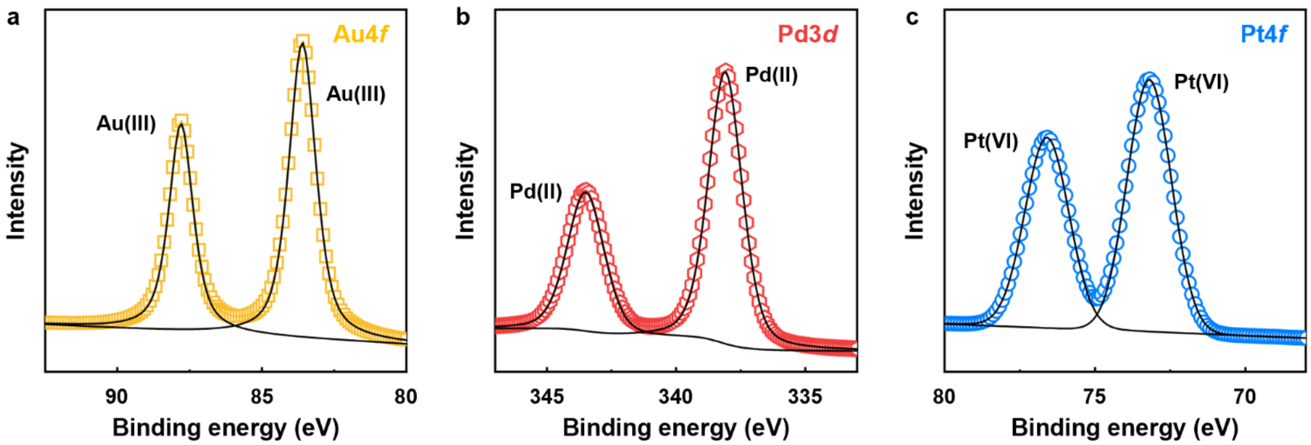

Supplementary Fig. 14. Deconvolution of the high-resolution (**a**) Au4*f*, (**b**) Pd3*d*, and (**c**) Pt4*f* XPS peaks of the PM/PAAm polymers. The PM/PAAm polymers were formed by allowing contact between PAAm (0.2 g L^{-1}) and PM (200 mg L^{-1}) aqueous solutions (pH = 2) for 3 h.

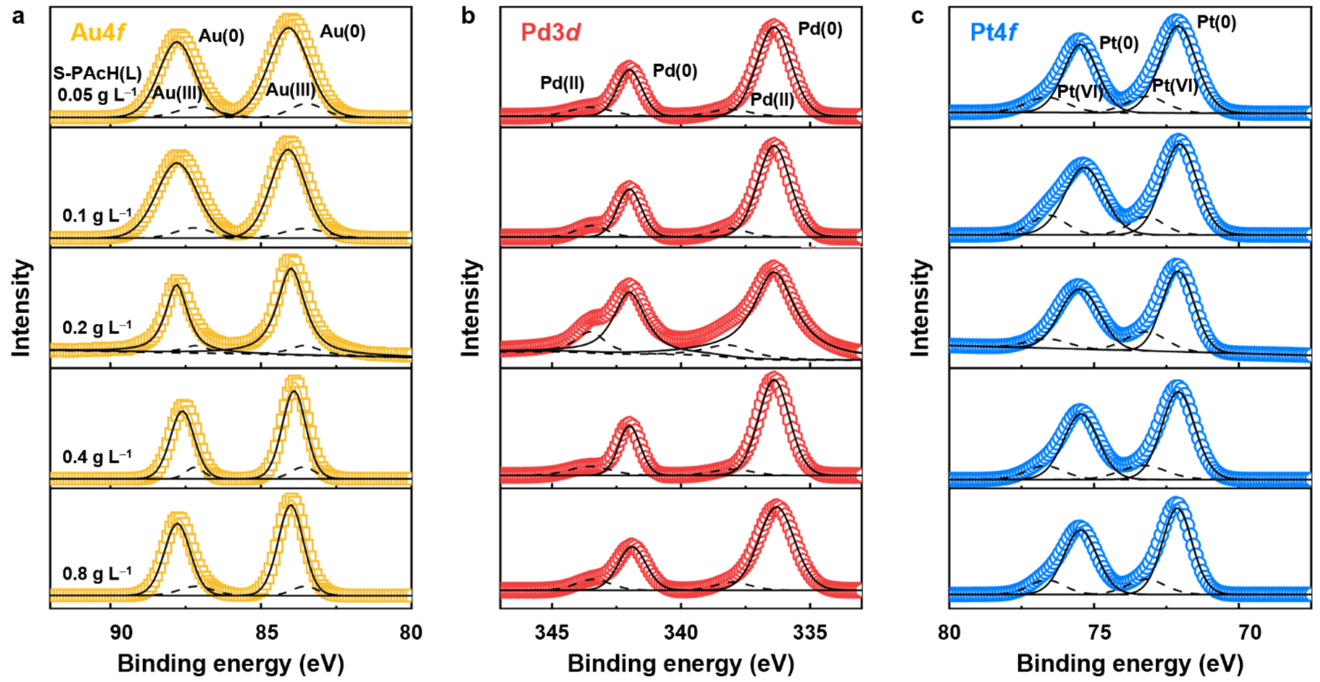

Supplementary Fig. 15. Deconvolution of the high-resolution (**a**) Au4*f*, (**b**) Pd3*d* and (**c**) Pt4*f* XPS peaks of the PM/S-PAcH(L) precipitates formed with different S-PAcH(L) concentrations. The precipitates were formed by allowing contact between S-PAcH(L) (0.05–0.8 g L^{-1}) and PM (200 mg L^{-1}) aqueous solutions (pH = 2) for 3 h.

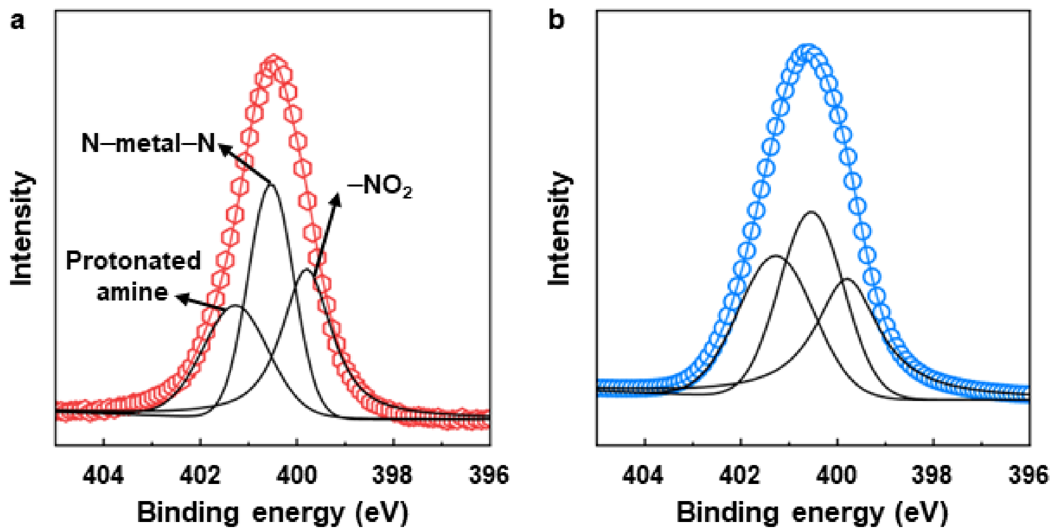

Supplementary Fig. 16. Deconvolution of the high-resolution N1*s* XPS spectra of the PM/S-PAcH(L) precipitates: (**a**) Pd and (**b**) Pt. The precipitates were formed by allowing contact between S-PAcH(L) (0.2 g L^{-1}) and PM (200 mg L^{-1}) aqueous solutions (pH = 2) for 3 h.

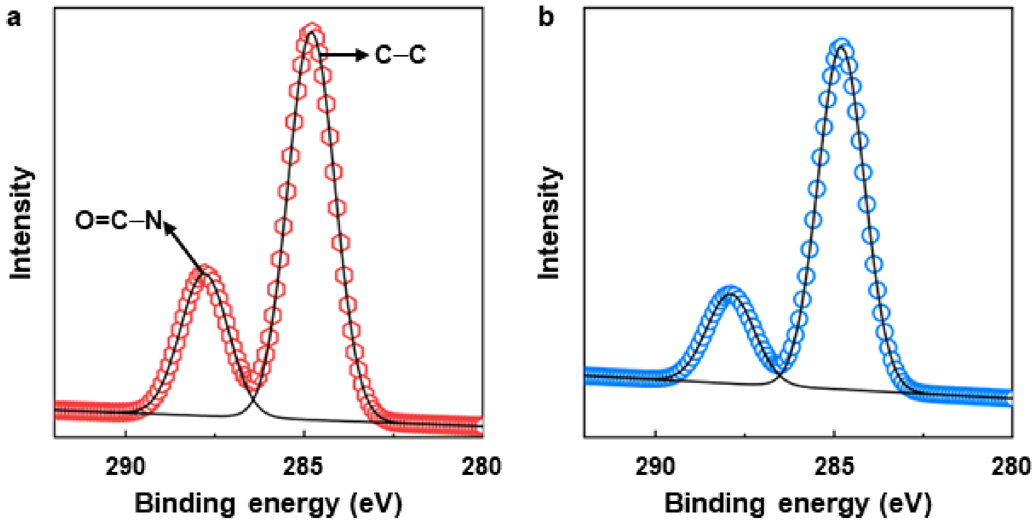

Supplementary Fig. 17. Deconvolution of the high-resolution C1*s* XPS spectra of the PM/S-PAcH(L) precipitates: (**a**) Pd and (**b**) Pt. The precipitates were formed by allowing contact between S-PAcH(L) (0.2 g L^{-1}) and PM (200 mg L^{-1}) aqueous solutions (pH = 2) for 3 h.

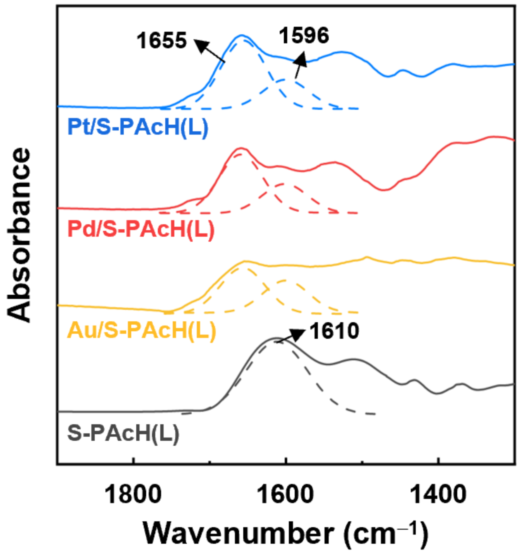

Supplementary Fig. 18. FT-IR spectra of the pristine S-PAcH(L) and PM/S-PAcH(L) precipitates. The precipitates were formed by allowing contact between S-PAcH(L) (0.2 g L^{-1}) and PM (200 mg L^{-1}) aqueous solutions (pH = 2) for 3 h. While pristine S-PAcH(L) exhibited the FT-IR peak at 1610 cm^{-1} (C=O stretching, amide)[22], all PM/S-PAcH(L)s displayed peaks at 1655 (C=O stretching, amide) and 1596 cm^{-1} (N=O stretching, $-NO_2$)[23]. It should be noted that the C=O stretching peak of PM/S-PAcH(L) shifted toward a longer wavenumber compared to that of S-PAcH(L) because the electron-withdrawing $-NO_2$ group of PM/S-PAcH(L) rendered the carbon atom of the C=O group electron-deficient[24].

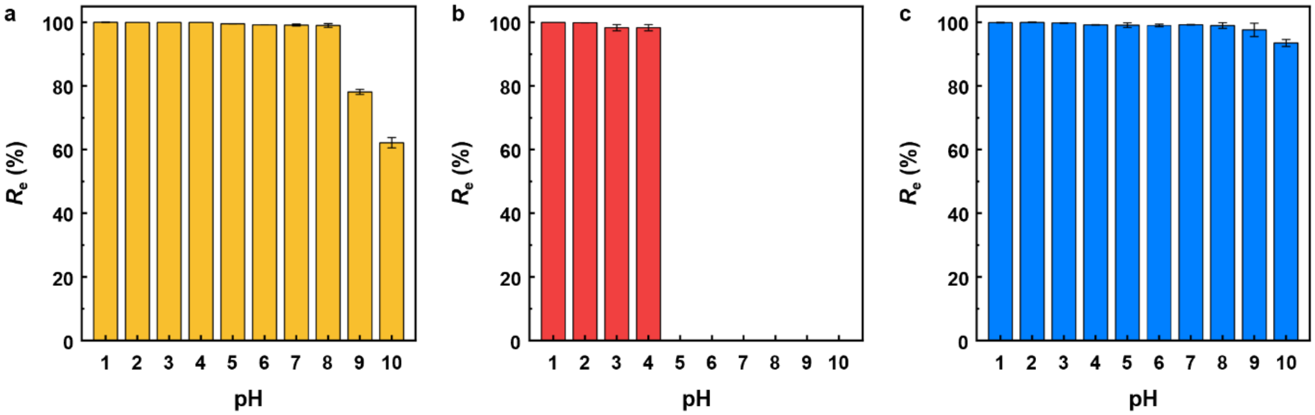

Supplementary Fig. 19. Recovery efficiency (R_e) of S-PAcH(L) for (**a**) Au, (**b**) Pd, and (**c**) Pt as a function of solution pH (S-PAcH(L) concentration = 0.2 g L^{-1}, C_i = 50 mg L^{-1}, contact time = 3 h). Error bars represent standard deviations determined from three replicates.

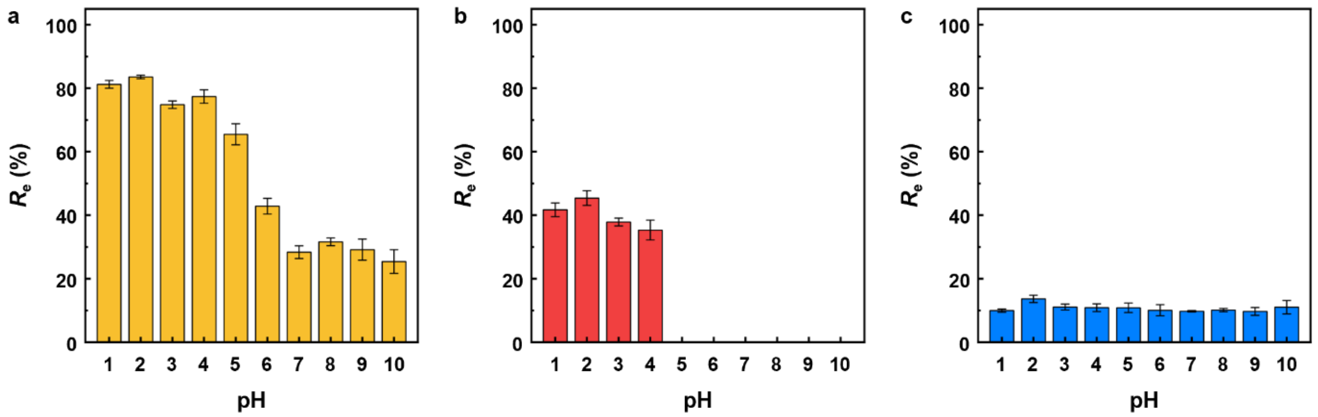

Supplementary Fig. 20. Recovery efficiency (R_e) of bPEI for (**a**) Au, (**b**) Pd, and (**c**) Pt as a function of solution pH (bPEI concentration = 0.2 g L^{-1}, C_i = 50 mg L^{-1}, contact time = 3 h). Error bars represent standard deviations determined from three replicates.

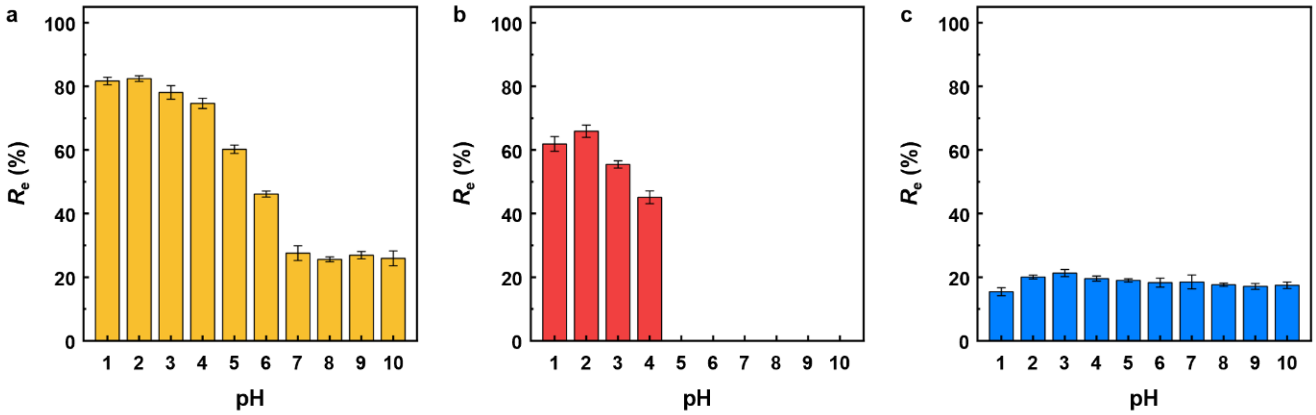

Supplementary Fig. 21. Recovery efficiency (R_e) of PAAm for (**a**) Au, (**b**) Pd, and (**c**) Pt as a function of solution pH (PAAm concentration = 0.2 g L^{-1}, C_i = 50 mg L^{-1}, contact time = 3 h). Error bars represent standard deviations determined from three replicates.

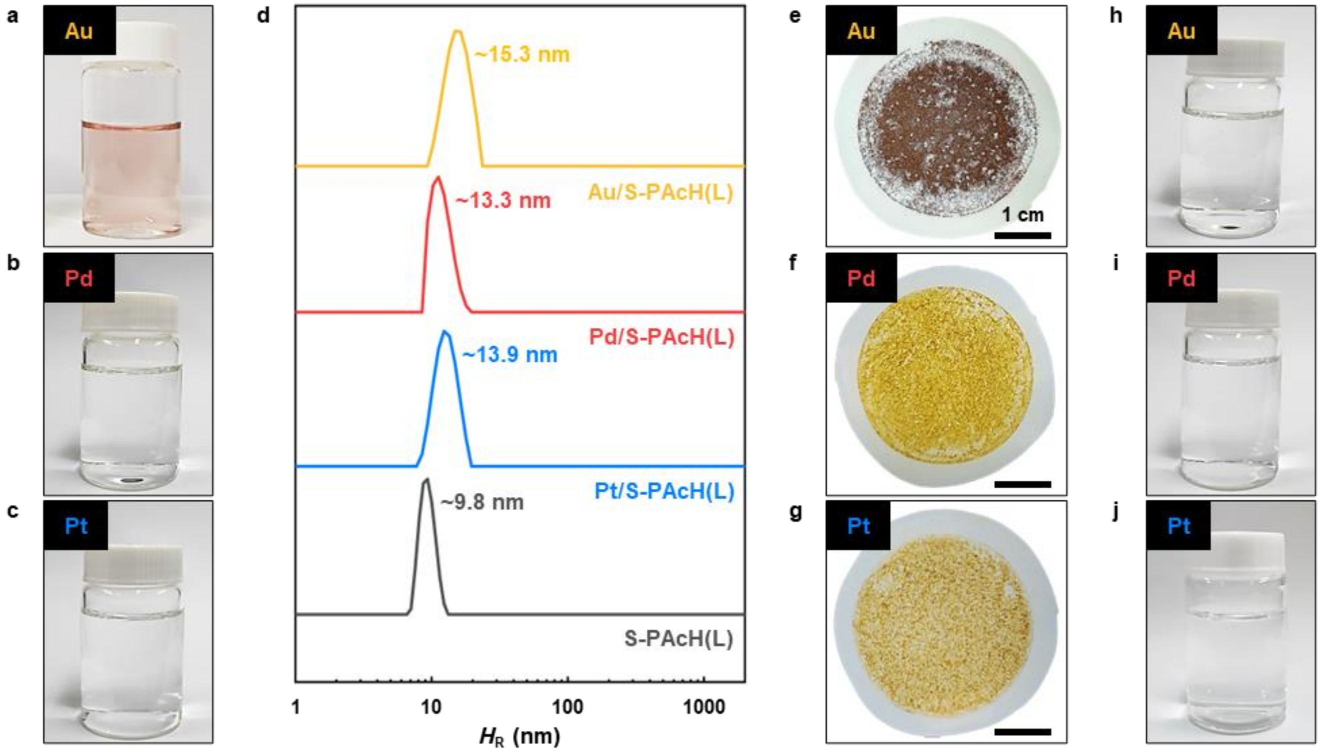

Supplementary Fig. 22. a–c, Photographs of the (**a**) Au, (**b**) Pd, and (**c**) Pt aqueous solutions after the addition of S-PAcH(L) (S-PAcH(L) concentration = 0.2 g L^{-1}, C_i = 1 mg L^{-1}, solution pH = 2, contact time = 3 h). **d,** Corresponding DLS curves of the pristine S-PAcH(L) and PM/S-PAcH(L) aqueous solutions. **e–j,** Photographs of the (**e–g**) PM/S-PAcH(L) particles screened by a PSF membrane and (**h–j**) permeate solutions: (**e, h**) Au, (**f, i**) Pd, and (**g, j**) Pt. Although microscale precipitates were not formed after the addition of S-PAcH(L) to the dilute PM solutions, a distinct increase in the hydrodynamic diameter (H_R) of S-PAcH(L) was observed. Moreover, the S-PAcH(L)-added Au solution exhibited the characteristic red color of Au NPs, indicating PM reduction by S-PAcH(L). Despite their small sizes, PM/S-PAcH(L) particles were completely screened by a PSF membrane (molecular weight cut-off = 20 kg mol^{-1}), as evidenced by PM/S-PAcH(L) particles collected by the membrane and clear permeate solutions. This result clearly demonstrates that even without microscale precipitation, a strong reduction mechanism enabled by S-PAcH(L) can achieve high R_e for all PMs at low C_i (<50 mg L^{-1}).

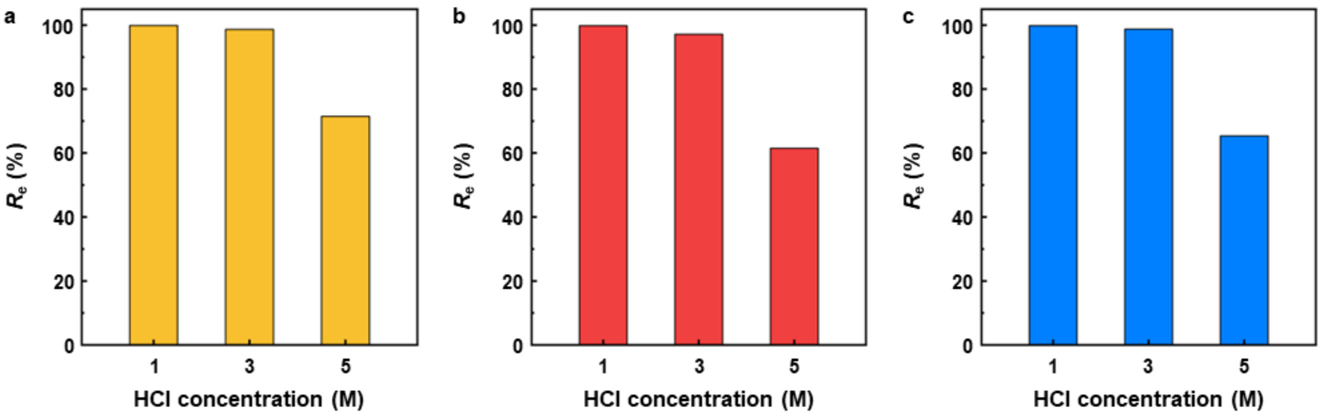

Supplementary Fig. 23. Recovery efficiency (R_e) of S-PAcH(L) for (**a**) Au, (**b**) Pd, and (**c**) Pt as a function of solution HCl concentrations (S-PAcH(L) concentration = 0.2 g L^{-1}, C_i = 50 mg L^{-1}, contact time = 3 h).

a

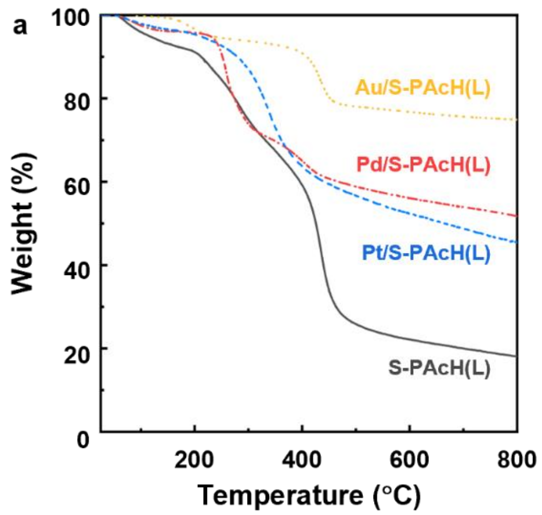

b

	$q_{e,TGA}$ (mg g^{-1})
Au	2265
Pd	696
Pt	502

Supplementary Fig. 24. a, TGA curves of pristine S-PAcH(L) and the PM/S-PAcH(L) precipitates. The precipitates were formed by allowing contact between S-PAcH(L) (0.2 g L^{-1}) and PM (800 mg L^{-1}) aqueous solutions (pH = 2) for 3 h. **b**, Amount of PM adsorbed onto S-PAcH(L), as calculated from the TGA analysis ($q_{e,TGA}$).

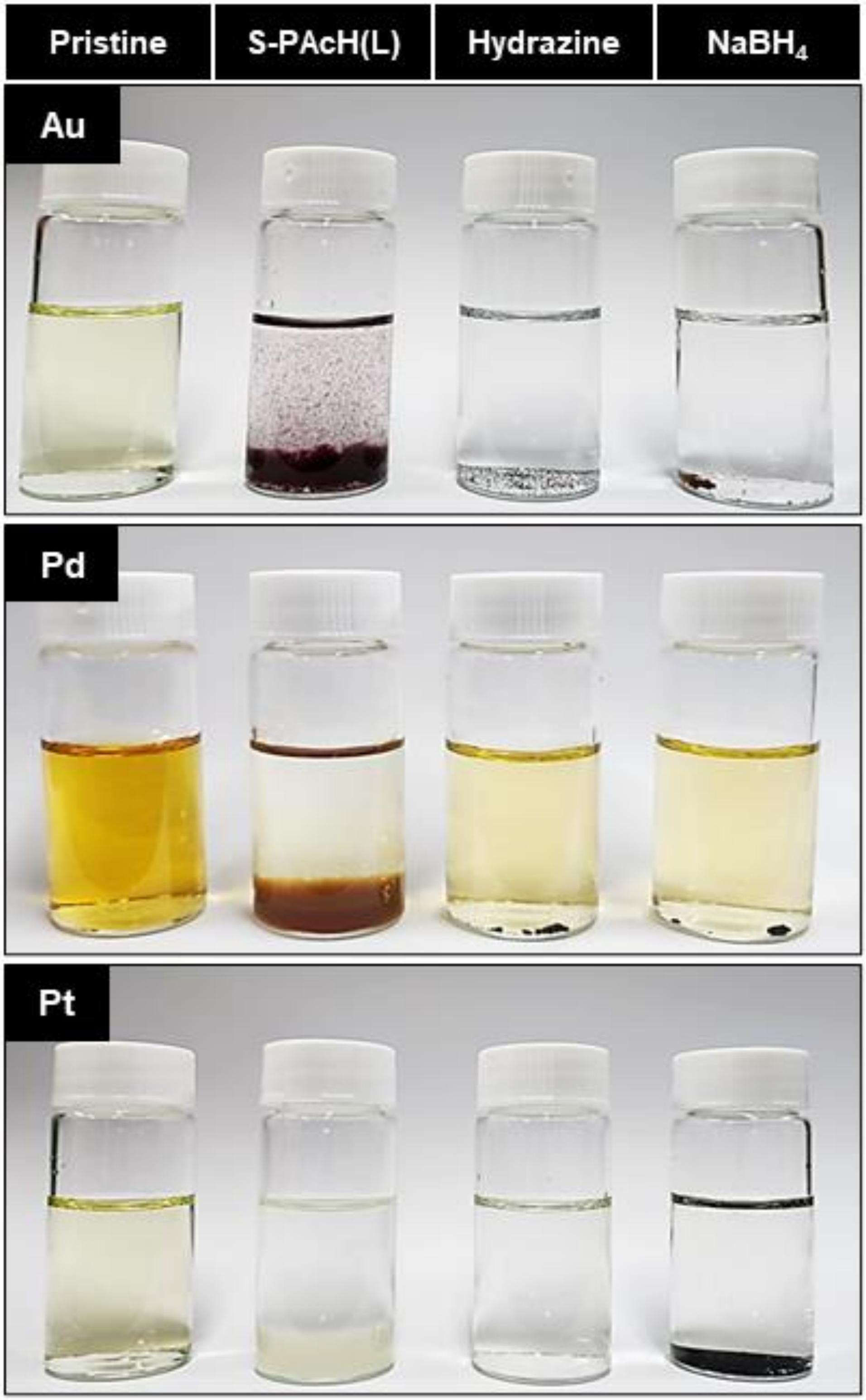

Supplementary Fig. 25. Photographs of the PM (200 mg L^{-1}) aqueous solutions after the addition of S-PAcH(L) and reducing agents (hydrazine and $NaBH_4$) (S-PAcH(L) and reducing agent concentration = 0.2 g L^{-1}, solution pH = 2, contact time = 3 h).

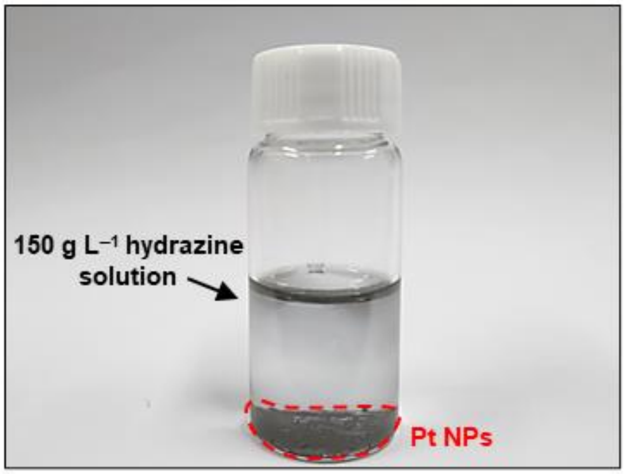

Supplementary Fig. 26. Photograph of the Pt (200 mg L^{-1}) aqueous solution after the addition of concentrated hydrazine (hydrazine concentration = 150 g L^{-1}, solution pH = 2, contact time = 3 h).

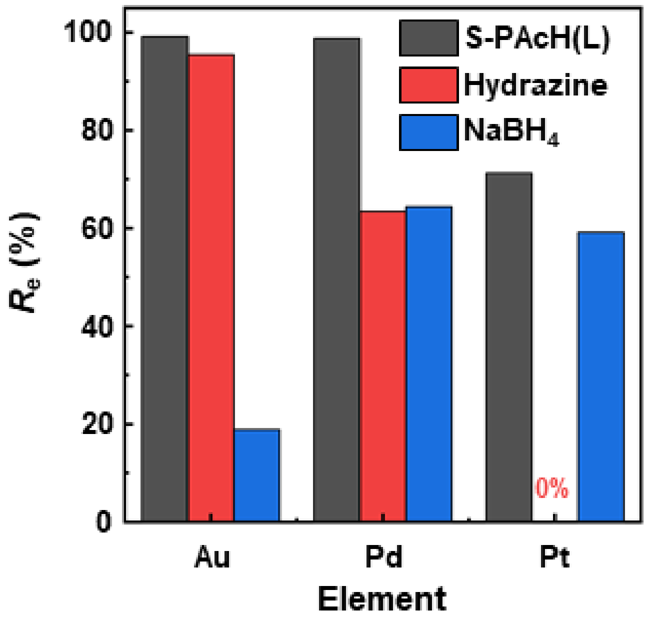

Supplementary Fig. 27. Recovery efficiency (R_e) of S-PAcH(L) and reducing agents (hydrazine and $NaBH_4$) for Au, Pd, and Pt (S-PAcH(L) and reducing agent concentration = 0.2 g L^{-1}, C_i = 200 mg L^{-1}, solution pH = 2, contact time = 3 h).

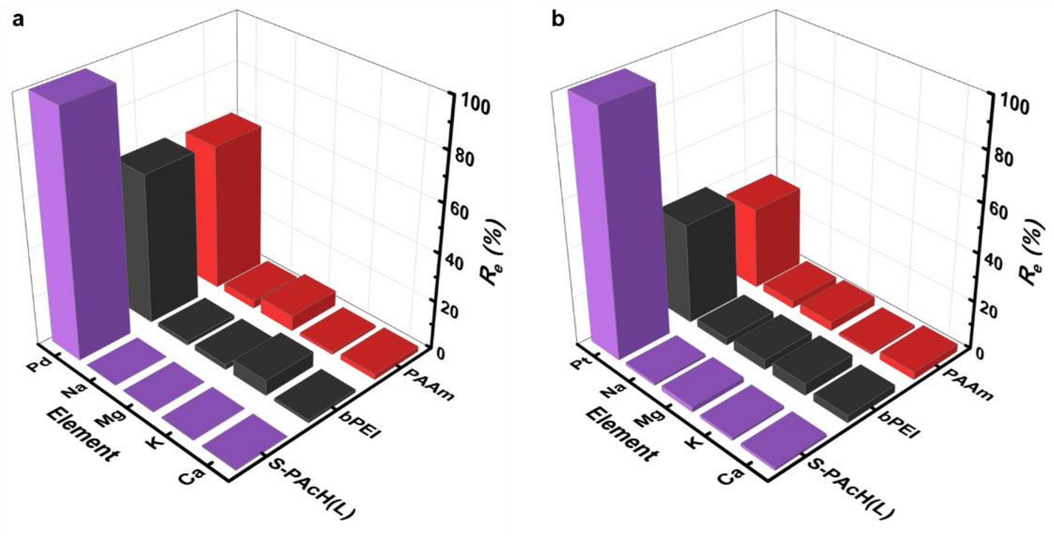

Supplementary Fig. 28. Recovery efficiency (R_e) of the polymers (S-PAcH(L), bPEI, and PAAm) with (**a**) Pd- and (**b**) Pt-containing simulated groundwater.

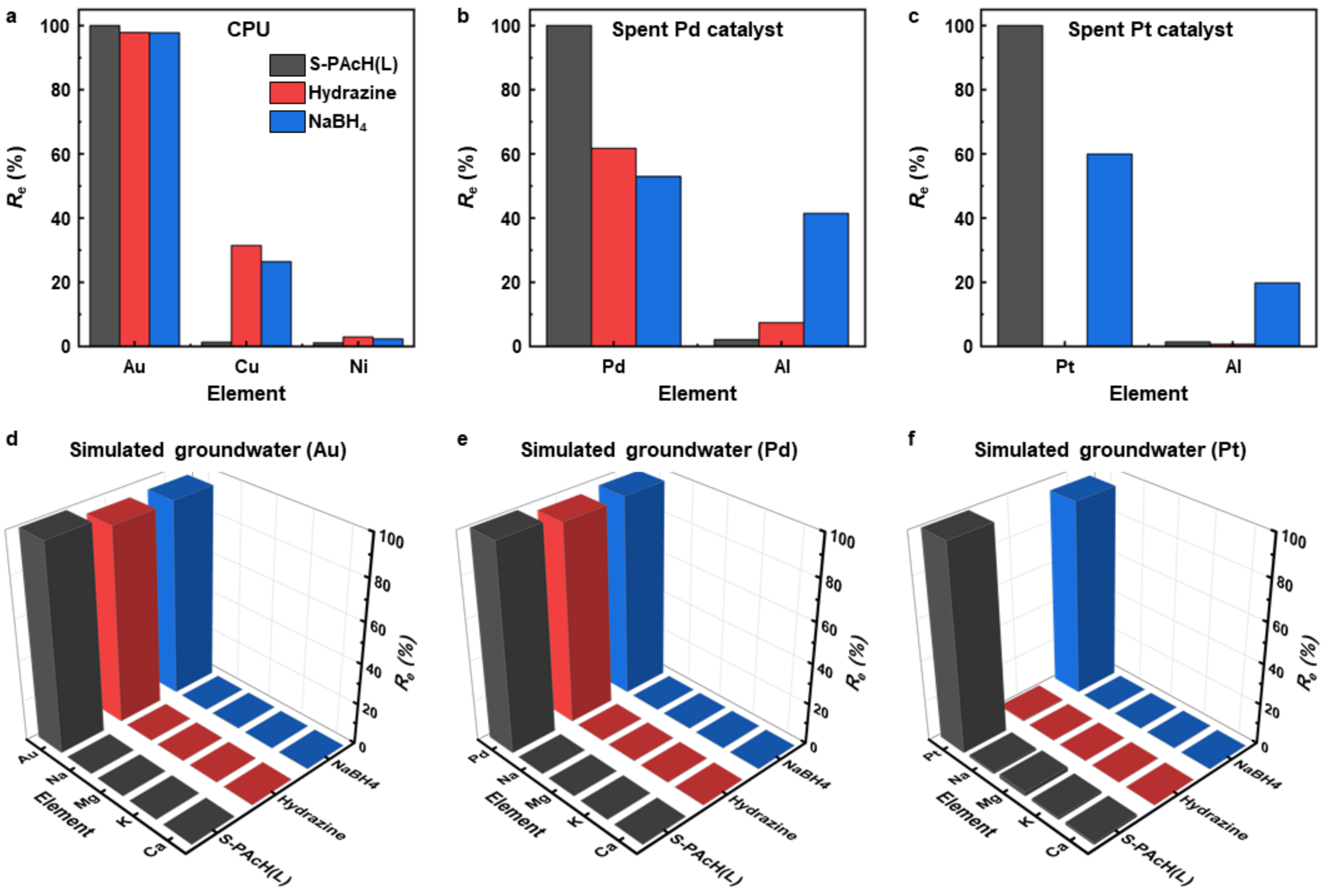

Supplementary Fig. 29. Recovery efficiency (R_e) of S-PAcH(L) and reducing agents (hydrazine and $NaBH_4$) with simulated leachate and groundwater feed solutions: (**a**) CPU leachate (Au), (**b**) spent Pd catalyst leachate, (**c**) spent Pt catalyst leachate, and (**d–f**) groundwater containing (**d**) Au, (**e**) Pd, and (**f**) Pt (polymer and reducing agent concentration = 0.2 g L^{-1}, solution pH = 2, contact time = 3 h).

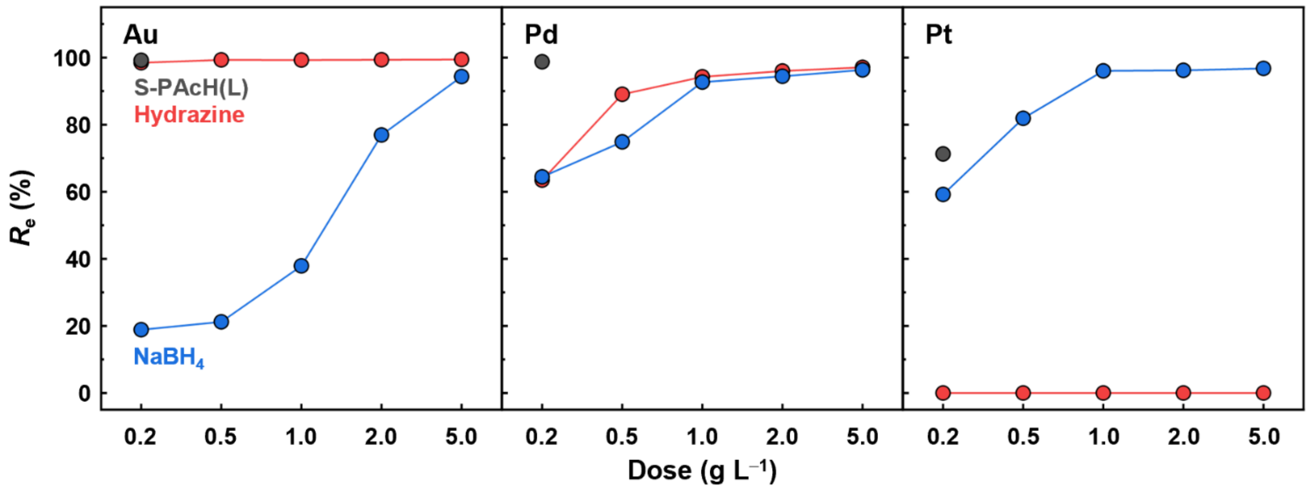

Supplementary Fig. 30. PM recovery efficiency (R_e) of S-PAcH(L) (0.2 g L^{-1}) and reducing agents (hydrazine and $NaBH_4$) as a function of reducing agent doses (C_i = 200 mg L^{-1}, solution pH = 2, contact time = 3 h).

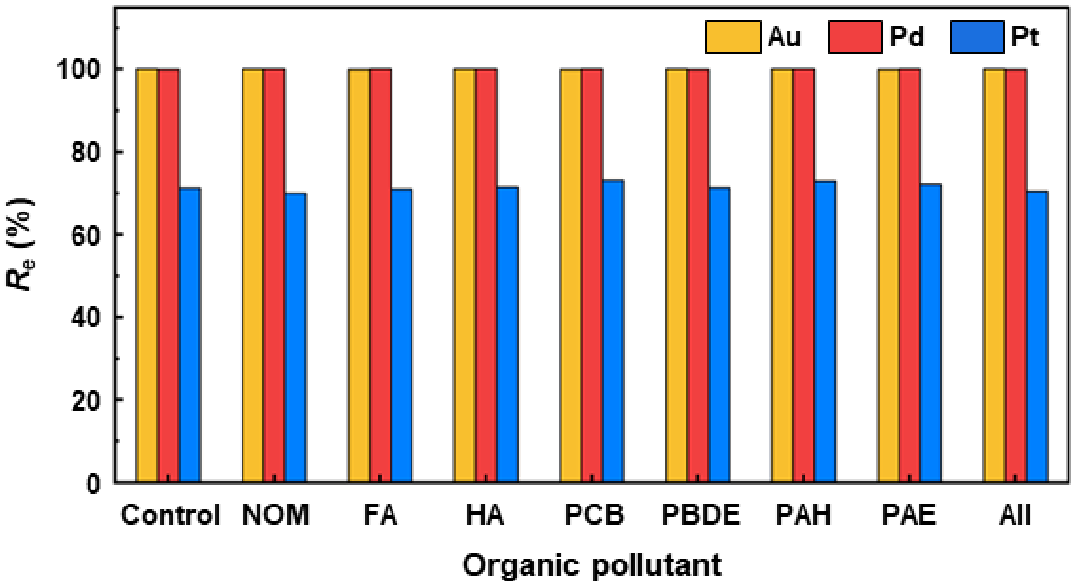

Supplementary Fig. 31. PM recovery efficiency (R_e) of S-PAcH(L) for Au, Pd, and Pt for PM aqueous solutions with and without (control) organic pollutants (S-PAcH(L) concentration = 0.2 g L^{-1}, C_i = 200 mg L^{-1}, organic pollutant concentration = 200 mg L^{-1} each, solution pH = 2, contact time = 3 h).

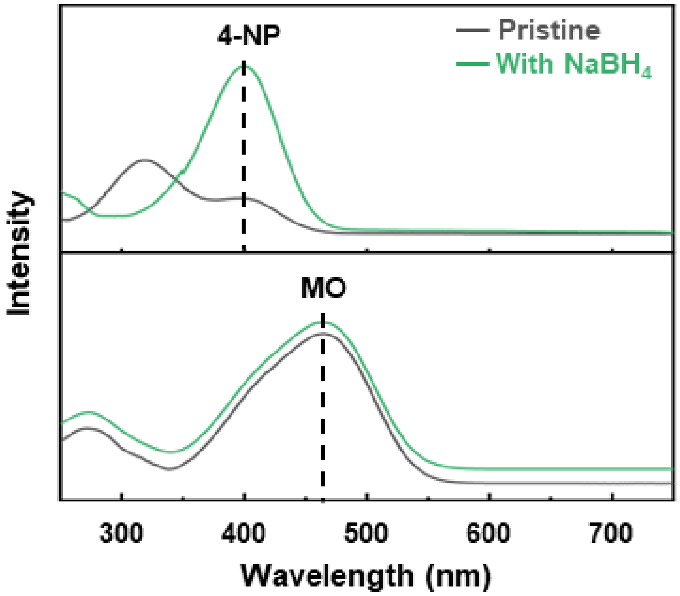

Supplementary Fig. 32. UV-vis spectra of organic dye (MO and 4-NP) solutions without (pristine) and with $NaBH_4$. In the presence of $NaBH_4$, the characteristic UV-vis peak of 4-NP shifted from 317 to 400 nm owing to the formation of 4-nitrophenolate[25], whereas that of MO was maintained at 464 nm.

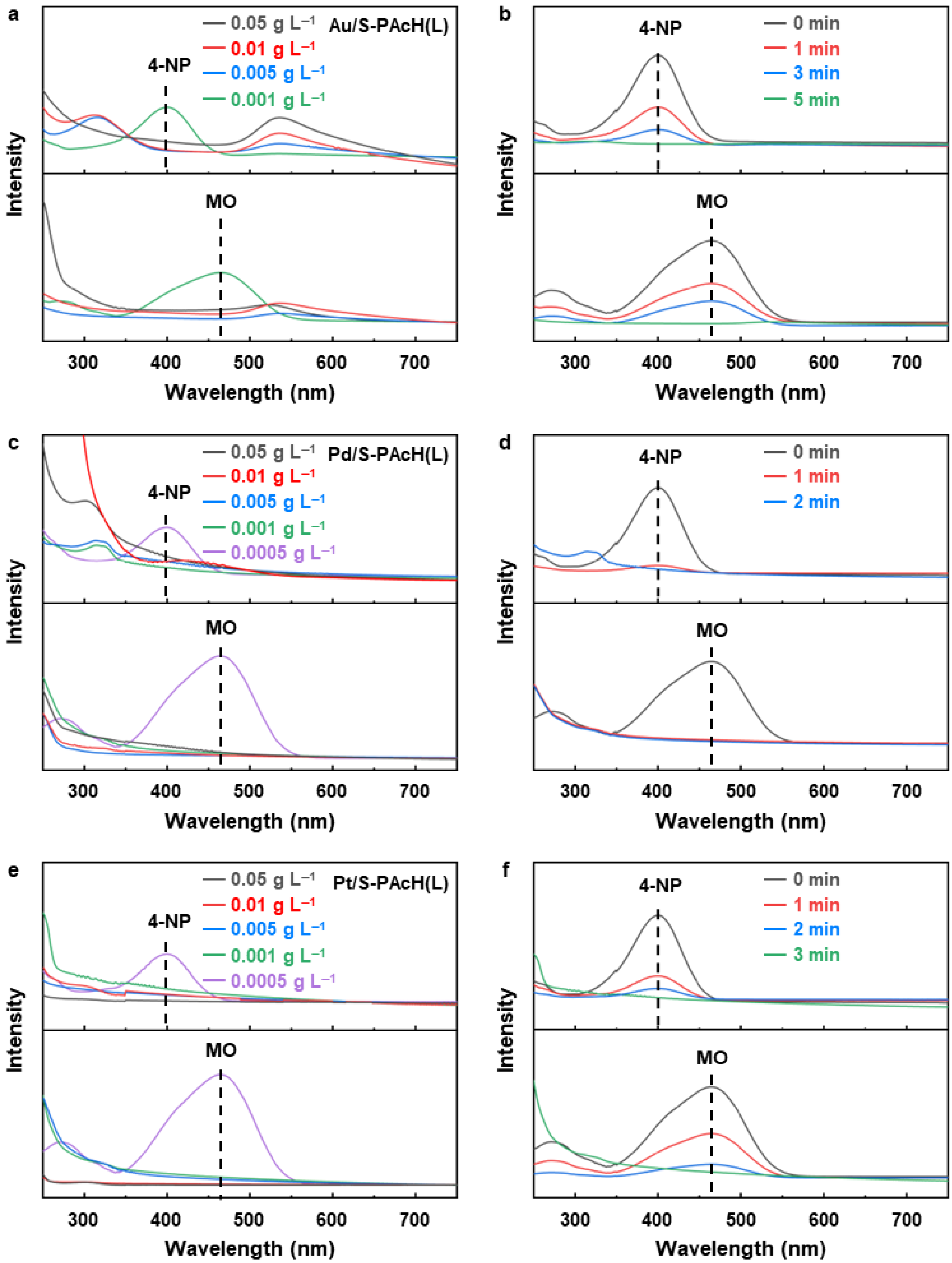

Supplementary Fig. 33. UV-vis spectra of organic dye (MO and 4-NP) solutions containing $NaBH_4$ after the addition of the Pt/S-PAcH(L) precipitates as a function of (**a, c, e**) PM/S-PAcH(L) concentrations (reaction time = 30 min) and (**b, d, f**) reaction times (Au/S-PAcH(L) concentration = 0.005 g L^{-1} and Pd/ and Pt/S-PAcH(L) concentration = 0.001 g L^{-1}): (**a, b**) Au/S-PAcH(L), (**c, d**) Pd/S-PAcH(L), and (**e, f**) Pt/S-PAcH(L).

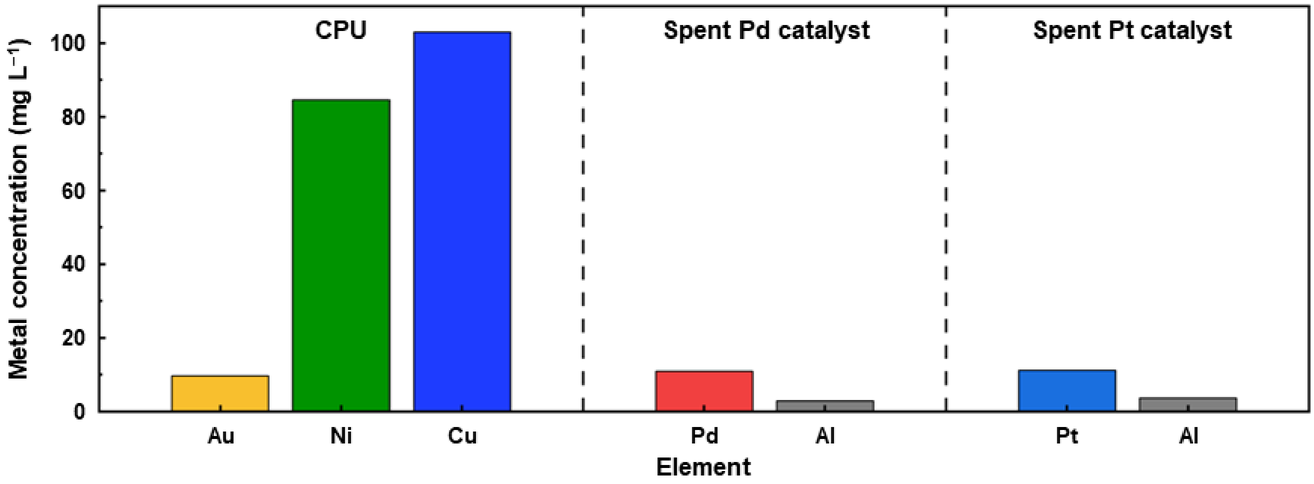

Supplementary Fig. 34. Metal composition of the real-world CPU and spent Pd and Pt catalyst leachates.

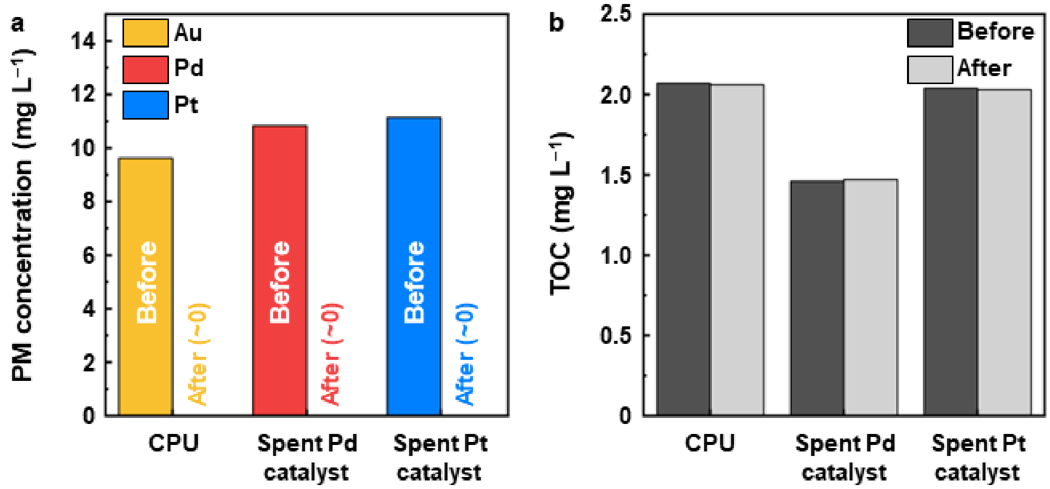

Supplementary Fig. 35. (**a**) PM ion and (**b**) TOC concentrations in the real-world CPU and spent catalyst (Pd and Pt) leachates before and after the addition of S-PAcH(L) (S-PAcH(L) concentration = 0.2 g L^{-1}, solution pH = 2, contact time = 3 h).

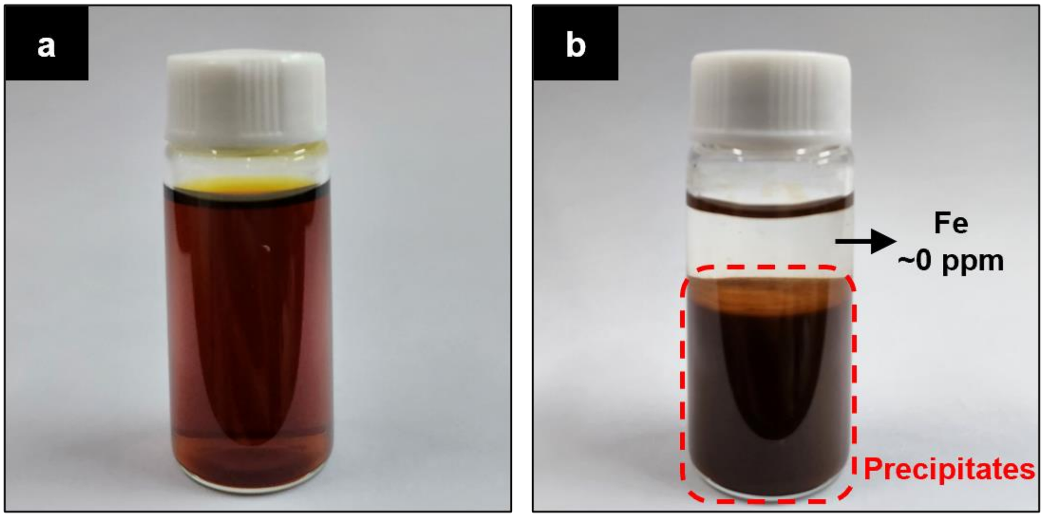

Supplementary Fig. 36. Photographs of the PM desorption aqueous solution containing thiourea (1M), $FeCl_3$ (1M), and HCl (1M) (**a**) before and (**b**) after adjusting the solution pH to 3. The pristine desorption solution is brown-colored (**a**). When the solution pH was adjusted to 3 using 30% ammonia solution (Daejung Chemical), brown-colored insoluble Fe precipitates and colorless supernatant solution were obtained (**b**). The precipitates were readily collected with a cellulose filter paper (pore size = 1 μm), and the Fe concentration in the permeate solution was measured to be ~0 ppm using ICP-OES. This result suggests that Fe ions can be readily removed by simple pH adjustment.

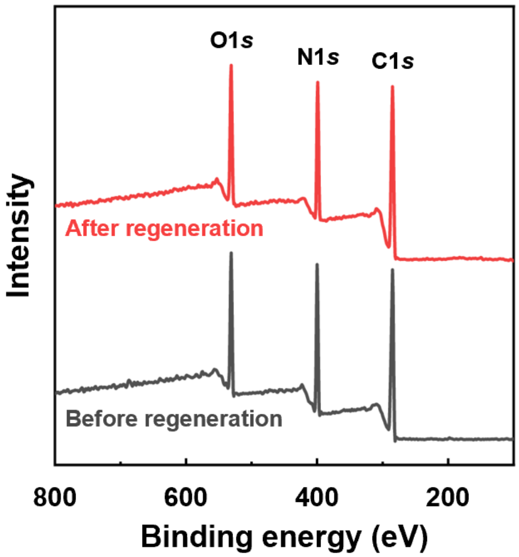

Supplementary Fig. 37. XPS survey spectra of S-PAcH(L) before and after the regeneration step. No S (corresponding to thiourea), Fe (corresponding to $FeCl_3$), Cl (corresponding to $FeCl_3$ and HCl), and PM peaks were detected for S-PAcH(L) before and after regeneration. This indicates that desorption agents and PM ions were completely removed from S-PAcH(L) by the regeneration process.

Supplementary Table 1. Structures and properties of the polymer adsorbents used in this study.

Polymer	Structure	M_w (kg mol^{-1})	Degree of polymerization	H_R (nm)
bPEI	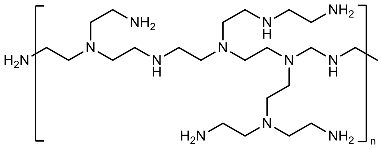	70	1625	13.6 ± 0.2
PAAm	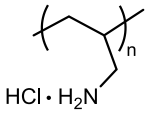	150	1603	15.4 ± 1.0
L-PAcH	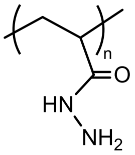	145	1684	16.8 ± 0.8
S-PAcH(s)	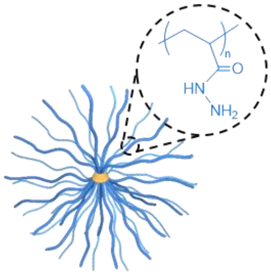	144	1627	6.0 ± 0.5
S-PAcH(L)		318	3648	9.8 ± 0.4

Supplementary Table 2. Binding energies and area percentages of the deconvoluted PM XPS peaks (Au4*f*, Pd3*d*, and Pt4*d*) of the PM/S-PAcH(L) precipitates.

Sample	Peak	Binding energy (eV)	Peak area percentage (%)	Peak assignment
Au/S-PAcH(L)	Au4*f*	84.0/87.8	88.8	Au(0)
		83.6/87.2	11.2	Au(III)
Pd/S-PAcH(L)	Pd3*d*	336.4/342.0	86.7	Pd(0)
		338.1/343.5	13.3	Pd(II)
Pd/S-PAcH(L)	Pt4*f*	72.1/75.5	80.6	Pt(0)
		73.2/76.7	19.4	Pt(VI)

Supplementary Table 3. Area percentages of reduced metal state (PM(0)) peaks in the deconvoluted PM XPS peaks (Au4*f*, Pd3*d*, and Pt4*f*) of the PM/S-PAcH(L) precipitates formed with different S-PAcH(L) concentrations.

S-PAcH(L) concentration (mg L^{-1})	Peak area percentage of PM(0) (%)		
	Au	Pd	Pt
0.05	88.9	86.5	80.1
0.1	88.9	86.4	80.6
0.2	88.8	86.7	80.6
0.4	88.4	86.7	80.5
0.8	88.5	86.5	80.2

Supplementary Table 4. Isotherm model fitting parameters of S-PAcH(L) for three PMs.

	Langmuir			Freundlich			Redlich-Peterson			
	q_m (mg g^{-1})	K_L (L mg^{-1})	R^2	n	K_F (mg g^{-1})	R^2	K_R (L mg^{-1})	α	a_R (mg L^{-1})	R^2
Au	2847	0.69	0.97	4.68	1030	0.84	1845	1.00	0.58	0.95
Pd	1078	20.52	0.80	12.30	718	0.73	2246	1.00	20.64	0.84
Pt	714	3.24	0.94	7.56	380	0.68	1000	1.00	1.38	0.82

Supplementary Table 5. Kinetics model fitting parameters of S-PAcH(L) for three PMs.

	Pseudo-first-order			Pseudo-second-order		
	q_e (mg g^{-1})	k_1 (min^{-1})	R^2	q_e (mg g^{-1})	k_2 (g mg^{-1} min^{-1})	R^2
Au	2821	2842	1.00	2821	0.47	1.00
Pd	1042	1106	1.00	1042	1.09×10^{21}	1.00
Pt	702	708	1.00	702	3.41	1.00

Supplementary Table 6. Adsorbent type, maximum adsorption capacity (q_m), equilibrium time, and proposed isotherm/kinetics models of S-PAcH(L) and other reported PM adsorbents.

Adsorbent type	Adsorbent	q_m (mg g^{-1})/equilibrium time[a] (min)			Isotherm model	Kinetics model	Ref.
		Au	Pd	Pt			
Metal-based adsorbents	Plant tannin-immobilized Fe_3O_4@SiO_2 microsphere	917/1440	196/1440	–	Langmuir	PSO[b]	(26)
	Nanometer-sized titanium dioxide	23/10	12/30	–	–	–	(27)
	Si-TpAL[c]	–	48/300	–	Langmuir	PSO	(28)
	Modified $CoFe_2O_4$	120/240	–	–	Langmuir	PSO	(29)
	MNP-G3[d]	4/480	3/480	–	Freundlich	PSO	(30)
	AHPP-MOF[e]	–	284/420	–	Langmuir	PSO	(31)
	Fe_3O_4 nanoparticle	–	11/180	13/180	–	–	(32)
	Thiol-modified mesoporous silica	–	10/300	18/480	–	–	(33)
	MPVA[f]	1425/10	–	–	Langmuir	PSO	(34)
	Magnetic alginate polymer-imprinted diatomite composite	–	60/140	–	Sips	PSO	(35)
	1,4,7,10-Tetraazacyclododecane-modified mesoporous silica	–	162/180	–	Langmuir	PSO	(36)
MOF[g]-based adsorbents	Fe-BTC[h]	934/2	–	–	–	–	(37)
	Zr(IV)-based MOF	280/25	120/25	166/25	Langmuir	–	(38)
	Thiourea-modified zirconium-based MOF	326/180	–	–	–	PSO	(39)
	MIL-101(Cr)-NH_2[i]	–	278/720	141/720	Langmuir	PSO	(40)
	UiO-66-BTU[j]	658/300	–	–	Langmuir	PSO	(41)
	MOF-AFH[k]	403/180	193/180	–	Langmuir	PSO	(42)
	UiO-66-NH_2[l]	650/1440	–	–	Langmuir	PSO	(43)
	CN-activated cobalt hexacyanoferrate	–	–	25/1440	Langmuir	PSO	(44)
	MIL-101(Cr)/ED-GA[m]	–	323/120	416/60	Langmuir	PSO	(45)
	PCN-222-MBA[n]	714/1	–	–	Langmuir	–	(46)
	IPMIL-101(Cr)[o]	–	193/40	–	Langmuir	PSO	(47)

Polymer-based adsorbents	PEPEI[p]	743/1080	509/1080	572/1080	Langmuir	–	(48)
	Poly(allylamine hydrochloride)-modified *E.coli*	–	–	304/45	Langmuir	PSO	(49)
	Tannic acid-coated porous poly(EGDMA-co-AN)[q] microsphere	52/60	–	–	Langmuir	PSO	(50)
	Chitosan-dibenzo-18-crown-6-ether	–	19/180	23/180	Sips	PSO	(51)
	poly-Cys-g-PDA@GPUF[r]	1083/30	785/30	574/30	Langmuir	–	(52)
	Amine-rich polymeric capsule	576/180	–	–	Langmuir	PSO	(53)
	Polyamine chelating resin	–	–	162/1200	Langmuir	PSO	(54)
	S-PAcH(L)	2847/1	1078/1	714/1	Langmuir	–	This study
Biomass-based adsorbents	Glycine-modified crosslinked chitosan resin	170/120	120/120	122/180	Langmuir	PSO	(10)
	Dimethylaniline-modified paper	906/300	224/300	176/300	Langmuir	–	(55)
	Human hair	638/1440	–	–	Langmuir	–	(56)
	N-aminoguanidine-modified persimmon tannin	1753/240	214/120	197/1440	Langmuir	–	(57)
	Chitosan grafted with 8-hydroxyquinoline-2-carbaldehyde	–	340/120	204/120	Langmuir	PSO	(58)
	PEI-incorporated algal bead	–	136/1440	115/360	Langmuir	PSO	(59)
	Thiourea-modified chitosan microsphere	–	42/150	57/300	–	PSO	(60)
	Thiourea-modified chitosan-imprinted resin	933/240	–	–	Langmuir	PSO	(61)
Carbon-based adsorbents	Poly acryloyl hydrazide-brushed CNT membrane	292/180	187/180	267/180	–	–	(12)
	Graphene oxide	108/360	81/120	71/40	Langmuir	PSO	(62)
	Mesoporous carbon	492/1080	64/1080	78/1080	–	–	(63)
	Graphene oxide-TOABr[s]	–	93/30	–	Langmuir	–	(64)
	B-N-WSBP biochar[t]	461/180	134/180	47/180	Langmuir	PSO	(65)
	Graphene oxide/calcium alginate hydrogel	82/1320	–	–	Langmuir	PSO	(66)
	Bismuth carbonate supported over carbon black	13/60	–	–	Sips	PSO	(67)

[a] Equilibrium time was defined by the time when q_t reaches 98% of q_e. [b] Pseudo-second-order. [c] 3-aminopropyl-functionalized silica-gel. [d] Magnetic nanoparticle modified by third-generation dendrimer. [e] 4-amino-3-hydroxybenzoic acid-modified Zr-based metal-organic framework. [f] Ti_3C_2 MXene-polyhedral oligomeric silsesquioxane/V_2O_3@C aerogel. [g] Metal-organic framework. [h]

Fe_3O clusters interlinked by 1,3,5-benzenetricarboxylate ligands. [i] Cr-based MOF. [j] Zr-based MOF functionalized by bisthiourea. [k] 2,6-aminopyridine-grafted 3-formyl-4-hydroxybenzoic acid-based MOF. [l] Zr-based MOF. [m] Cr-based MOF functionalized with ethylenediamine-glutaraldehyde. [n] Porous coordination network with 4-mercaptobenzoic acid. [o] Pd^{2+} ion-imprinted Cr-based MOF. [p] *N*-2-(2-pyridyl)-ethylpolyethyleneimine. [q] Poly(ethylene glycol dimethacrylate-co-acrylonitrile). [r] Cysteine polymer brush-grafted polydopamine-modified graphene-based polyurethane foam. [s] Tetraoctylammonium bromide-impregnated graphene oxide. [t] Boron-nitrogen co-doped walnut shell biochar powder.

Supplementary Table 7. Unit prices, used amounts, and respective costs of the raw materials used for synthesizing S-PAcH(L) of 1 kg.

Raw material	Manufacturer	Unit price	Used amount	Cost
CDx	Sigma-Aldrich	\$997.4 kg^{-1}	3.6 g	\$3.6
BiBr	Sigma-Aldrich	\$409.4 kg^{-1}	24.5 g	\$10.0
CuBr	Sigma-Aldrich	\$3401 kg^{-1}	8.3 g	\$28.2
MAc	Sigma-Aldrich	\$52.8 L^{-1}	4.6 L	\$242.8
Alumina	Sigma-Aldrich	\$156.5 kg^{-1}	1.3 kg	\$195.6
TBABr	Sigma-Aldrich	\$1706.8 kg^{-1}	1.0 kg	\$1706.8
Hydrazine hydrate	Sigma-Aldrich	\$379.8 kg^{-1}	7.6 kg	\$2886.5
Na_2CO_3	Daejung Chemical	\$141.4 kg^{-1}	46.1 g	\$6.5
DCM	Daejung Chemical	\$6.8 L^{-1}	214.3 mL	\$1.5
THF	Daejung Chemical	\$21.2 L^{-1}	30.0 L	\$634.6
Methanol	Daejung Chemical	\$7.2 L^{-1}	162.5 L	\$1175
NMP	Daejung Chemical	\$89.7 L^{-1}	29.1 mL	\$2.6
Silica	Alfa Aesar	\$53.8 kg^{-1}	1.3 kg	\$67.3
DI water	Millipore	\$1.5 L^{-1}	214.3 mL	\$0.3
		Total cost:		\$6961.3 kg^{-1}
		Expected final price[a]:		\$7734.8 –11602.2 kg^{-1}

[a] Estimated with the assumption that raw material costs occupy approximately 60–90% of the total production cost[17].

Supplementary Table 8. Prices, required doses, and respective costs of the materials used for PM recovery.

Materials	Manufacturer	Material price ($ kg^{-1})	Dose (g L^{-1})			Cost ($ L^{-1})		
			Au	Pd	Pt	Au	Pd	Pt
S-PAcH(L)	Lab-made	7734.8 –11602.2	0.2	0.2	0.2	1.55–2.32	1.55–2.32	1.55–2.32
Hydrazine	Sigma-Aldrich	379.8	5.0	>5.0	>>5.0	1.90	>1.90	>>1.90
$NaBH_4$	Daejung Chemical	157.7	>5.0	>5.0	0.36	>0.79	>0.79	0.06

Supplementary Table 9. TON and TOF values of the catalysts for 4-NP reduction.

Catalyst	$C_{4\text{-NP}}$ (mM)	$NaBH_4$ concentration (mM)	C_{cat} (g L^{-1})	t (min)	TON (mmol g^{-1})	TOF (mmol g^{-1} min^{-1})	Ref.
Au/S-PAcH(L)	0.01	1	0.005	5	2	0.4	
Pd/S-PAcH(L)	0.01	1	0.001	2	10	5	This work
Pt/S-PAcH(L)	0.01	1	0.001	3	10	3.3	
Au NP	2	100	0.20	3.3	10.26	3.11	(68)
Au NP	0.05	13	0.08	5	0.60	0.12	(69)
APM-Au NP[a]	4	100	0.20	9	20.31	2.26	(70)
Au NP	2	30	0.20	10	10.15	1.01	(71)
Au NP	0.05	10	1.97	8.6	0.03	0.003	(72)
Pd NP	1.25	125	0.10	71	12.50	0.18	(73)
Pd NP@chitosan-MWCNT[b]	0.091	4.54	3.63	12	0.03	0.002	(74)
Pd NP@Sch-boehmite[c]	0.114	4.54	0.91	4	0.13	0.03	(75)
GG-s-Pt NP[d]	1	100	0.49	240	2.10	0.009	(76)
Pt NP	0.67	6.67	3.33	20	0.20	0.01	(77)

[a] Aspartam-capped Au NP. [b] Pd NP-loaded crosslinked chitosan/MWCNT bead. [c] Pd NP on Schiff base-modified boehmite. [d] Pt NP stabilized by guar gum.

Supplementary Table 10. TON and TOF values of the catalysts for MO reduction.

Catalyst	C_{MO} (mM)	$NaBH_4$ concentration (mM)	C_{cat} (g L^{-1})	*t* (min)	TON (mmol g^{-1})	TOF (mmol g^{-1} min^{-1})	Ref.
Au/S-PAcH(L)	0.01	1	0.005	5	2	0.4	
Pd/S-PAcH(L)	0.01	1	0.001	1	10	10	This work
Pt/S-PAcH(L)	0.01	1	0.001	3	10	3.3	
Pd NP@chitosan-MWCNT	0.1	79.3	4	1	0.025	0.025	(74)
DLP-Au NP[a]	0.1	79.3	4	8	0.025	0.003	(78)
MgAlCe-LDH@Au[b]	0.08	16	0.007	3.5	12.4	3.6	(79)
MA@Ag NP[c]	0.04	0.17	6.7	16	0.006	0.004	(80)
MA@Cu NP[d]	0.04	0.17	6.7	12	0.006	0.005	(80)
CuO-MgO	0.05	5	0.2	15	0.25	0.017	(81)
Cu-NMOF/Ce-doped-Mg-Al-LDH[e]	0.07	8.4	0.017	1	4	4	(82)
Cu-MOF[f]	0.07	8.4	0.017	3.5	4	1.1	(82)
Au/ZIF-11[g]	0.22	3.5	0.015	7	15.3	2.2	(83)

[a] Dalspinin-mediated AuNP. [b] Au NP-loaded Ce-doped magnesium-aluminium layered double hydroxide. [c] *M. azedarach*-supported Ag NP. [d] *M. azedarach*-supported Cu NP. [e] Copper-based nanoscale metal-organic framework combined with Ce-doped magnesium-aluminum layered double hydroxide. [f] Copper-based metal-organic framework. [g] Au NP-decorated ZIF-11.

Supplementary Table 11. Reductions in R_e per adsorption–desorption cycle of reported PM adsorbents.

Adsorbent	PM	Reduction in R_e per adsorption–desorption cycle	Ref.
ADH@BC hybrid membrane[a]	Au	0.4	(8)
MNP-G3[b]	Pd	1.7	(30)
AHPP-MOF[c]	Pd	2.1	(31)
MPVA[d]	Au	1.0	(34)
Magnetic alginate polymer-imprinted diatomite composite	Pd	2.9	(35)
1,4,7,10-Tetraazacyclododecane-modified mesoporous silica	Pd	1.2	(36)
CN-activated cobalt hexacyanoferrate	Pt	1.6	(44)
MIL-101(Cr)/ED-GA[e]	Pd	4.0	(45)
PCN-222-MBA[f]	Au	0.2	(46)
IPMIL-101(Cr)[g]	Pd	1.0	(47)
Poly(allylamine hydrochloride)-modified *E. coli*	Pt	4.4	(49)
Poly-Cys-*g*-PDA@GPUF[h]	Au	2.5	(52)
Polyamine chelating resin	Pt	4.0	(54)
Thiourea-modified chitosan-imprinted resin	Au	3.6	(61)
Bismuth carbonate supported over carbon black	Au	13.3	(67)
2-Mercaptobenzothiazole-impregnated amine-functionalized resin	Pd	4.8	(84)
	Au	0.7	
S-PAcH(L)	Pd	0.7	This study
	Pt	2	

[a] Adipic dihydrazide-grafted bacterial cellulose hybrid membrane. [b] Magnetic nanoparticle modified by third-generation dendrimer. [c] 4-amino-3-hydroxybenzoic acid-modified Zr-based metal-organic framework. [d] Ti_3C_2 MXene-polyhedral oligomeric silsesquioxane/V_2O_3@C aerogel. [e] Cr-based MOF functionalized with ethylenediamine-glutaraldehyde. [f] Porous coordination network with 4-mercaptobenzoic acid. [g] Pd^{2+} ion-imprinted Cr-based MOF. [h] Cysteine polymer brush-grafted polydopamine-modified graphene-based polyurethane foam.

Supplementary Table 12. Weight and cost ratios of adsorbed PM to S-PAcH(L) in PM/S-PAcH(L)s formed with different PM concentrations.

PM concentration	PM	Unit price of PM	Weight ratio of adsorbed PM to S-PAcH(L)	Price ratio of adsorbed PM to S-PAcH(L)
Model solution<br>PM: 200 mg L^{-1}	Au	\$65819 kg^{-1}	1	5.7–8.5
	Pd	\$33256 kg^{-1}	1	2.9–4.3
	Pt	\$31195 kg^{-1}	0.71	1.9–2.9
Real-world leachate<br>Au: 9.6 mg L^{-1}<br>Pd: 10.8 mg L^{-1}<br>Pt: 11.1 mg L^{-1}	Au	\$65819 kg^{-1}	0.048	0.27–0.41
	Pd	\$33256 kg^{-1}	0.054	0.16–0.23
	Pt	\$31195 kg^{-1}	0.056	0.15–0.22

Supplementary References

1. Al-Ghouti, M. A. & Da'ana, D. A. Guidelines for the use and interpretation of adsorption isotherm models: A review. *J. Hazard. Mater.* **393**, 122383 (2020).
2. Wang, J. & Guo, X. Adsorption kinetic models: Physical meanings, applications, and solving methods. *J. Hazard. Mater.* **390**, 122156 (2020).
3. Luo, M., Wang, Z., Zhang, C., Song, B., Li, D., Cao, P., Peng, X., Lui, S. & Liu, S. Advanced oxidation processes and selection of industrial water source: A new sight from natural organic matter. *Chemosphere* **303**, 135183 (2022).
4. Yu, F., Yang, C., Zhu, Z., Bai, X. & Ma, J. (2019). Adsorption behavior of organic pollutants and metals on micro/nanoplastics in the aquatic environment. *Sci. Total Environ.* **694**, 133643 (2019).
5. Costentin, C., Drouet, S., Robert, M. & Savéant, J.-M., Turnover numbers, turnover frequencies, and overpotential in molecular catalysis of electrochemical reactions. cyclic voltammetry and preparative-scale electrolysis. *J. Am. Chem. Soc.* **134**, 11235−11242 (2012).
6. Kästner, C. & Thünemann, A. F., Catalytic reduction of 4-Nitrophenol using silver nanoparticles with adjustable activity. *Langmuir* **32**, 7383−7391 (2016).
7. Saha, S., Pal, A., Kundu, S., Basu, S. & Pal, T., Photochemical green synthesis of calcium-alginate-stabilized Ag and Au nanoparticles and their catalytic application to 4-nitrophenol reduction. *Langmuir* **26**, 2885−2893 (2010).
8. Zhang, X., Li, H., Ye, M., Zhang, H., Wang, G. & Zhang, Y. Bacterial cellulose hybrid membrane grafted with high ratio of adipic dihydrazide for highly efficient and selective recovery of gold from e-waste. *Sep. Purif. Technol.* **292**, 121021 (2022).
9. Gurung, M., Adhikari, B. B., Alam, S., Kawakita, H., Ohto, K. & Inoue, K. Persimmon tannin-based new sorption material for resource recycling and recovery of precious metals. *Chem. Eng. J.* **228**, 405−414 (2013)
10. Ramesh, A., Hasegawa, H., Sugimoto, W., Maki, T. & Udea, K. Adsorption of gold(III), platinum(IV)

and palladium(II) onto glycine modified crosslinked chitosan resin. *Bioresour. Technol.* **99**, 3801–3809 (2008).

11. Park, J., Won, S. W., Mao, J., Kwak, I. S. & Yun, Y.-S. Recovery of Pd(II) from hydrochloric solution using polyallylamine hydrochloride-modified Escherichia coli biomass. *J. Hazard. Mater.* **181**, 794–800 (2010).
12. Zhang, L., Zha, X., Zhang, G., Gu, J., Zhang, W., Huang, Y., Zhang, J. & Chen, T. Designing a reductive hybrid membrane to selectively capture noble metallic ions during oil/water emulsion separation with further function enhancement. *J. Mater. Chem. A* **6**, 10217 (2018).
13. Wu, H., Wang, Y., Jones, L. O., Liu, W., Zhang, L., Song, B., Chen, X.-Y., Stern, C. L., Schatz, G. C. & Stoddart, J. F. (2021). Selective Separation of Hexachloroplatinate (IV) Dianions Based on Exo-Binding with Cucurbit [6] uril. *Angew. Chem. Int. Ed.* **60**, 17587–17594 (2021).
14. Lisiecki, I. & Pileni, M. P. Synthesis of copper metallic clusters using reverse micelles as microreactors. *J. Am. Chem. Soc.* **115**, 3887–3896 (1993).
15. Arvizo, R. R., Bhattacharyya, S., Kudgus, R. A., Giri, K., Bhattacharya, R. & Mukherjee, P. Intrinsic therapeutic applications of noble metal nanoparticles: past, present and future. *Chem. Soc. Rev.* **41**, 2943–2970 (2012).
16. Deraedt, C., Salmon, L., Gatard, S., Ciganda, R., Hernandez, R., Ruiz, J. & Astruc, D. Sodium borohydride stabilizes very active gold nanoparticle catalysts. *Chem. Commun.* **50**, 14194–14196 (2014).
17. Meneses, R. A. M., Cabrera-Papamija, G., Machuca-Martínez, F., Rodríguez, L. A., Diosa, J. E. & Mosquera-Vargas, E. Plastic recycling and their use as raw material for the synthesis of carbonaceous materials. *Heliyon* **8**, e09028 (2022) .
18. Chen, J. P. & Lim, L. L. Key factors in chemical reduction by hydrazine for recovery of precious metals. *Chemosphere* **49**, 363–370 (2002).
19. Biswas, F. B., Rahman, I. M., Nakakubo, K., Yunoshita, K., Endo, M., Nagai, K., Mashio, A. S.,

Taniguchi, T., Nishimura, T., Maeda, K. & Hasegawa, H. Selective recovery of silver and palladium from acidic waste solutions using dithiocarbamate-functionalized cellulose. *Chem. Eng. J.* **407**, 127225 (2021).

20. Yang, J., Kubota, F., Baba, Y., Kamiya, N. & Goto, M. Application of cellulose acetate to the selective adsorption and recovery of Au (III). *Carbohydr. Polym.* **111**, 768−774 (2014).
21. Pang, X., Zhao, L., Akinc, M., Kim, J. K. & Lin, Z. Novel amphiphilic multi-arm, star-like block copolymers as unimolecular micelles. *Macromolecules* **44**, 3746–3752 (2011).
22. Jeon, S., Park, C. H., Shin, S. S. & Lee, J.-H. Fabrication and structural tailoring of reverse osmosis membranes using *β*-cyclodextrin-cored star polymers. *J. Membr. Sci.* **611**, 118415 (2020).
23. Xavier, S. & Periandy, S. Spectroscopic (FT-IR, FT-Raman, UV and NMR) investigation on 1-phenyl-2-nitropropene by quantum computational calculations. *Spectrochim. Acta, Part A* **149**, 216–230 (2015).
24. Singh, J. S. FTIR and Raman spectra and fundamental frequencies of 5-halosubstituted uracils: 5-X-uracil (X= F, Cl, Br and I). *Spectrochim Acta, Part A* **87**, 106−111 (2012).
25. Ruan, M., Song, P., Liu, J., Li, E. & Xu, W. Highly efficient regeneration of deactivated Au/C catalyst for 4-nitrophenol reduction. *J. Phys. Chem. C* **121**, 25882–25887 (2017).
26. Fan, R., Min, H., Hong, X., Yi, Q., Liu, W., Zhang, Q. & Luo, Z. Plant tannin immobilized Fe_3O_4@SiO_2 microspheres: A novel and green magnetic bio-sorbent with superior adsorption capacities for gold and palladium. *J. Hazard. Mater* **364**, 780−790 (2019).
27. Qing, Y., Hang, Y., Wanjaul, R., Jiang, Z. & Hu, B. Adsorption behavior of noble metal ions (Au, Ag, Pd) on nanometer-size titanium dioxide with ICP-AES. *Anal. Sci.* **19**, 1417−1420 (2003).
28. Wu, H., Kim, S.-Y., Ito, T., Miwa, M. & Matsuyama, S. One-pot synthesis of silica-gel-based adsorbent with Schiff base group for the recovery of palladium ions from simulated high-level liquid waste. *Nucl. Eng. Technol.* **54**, 3641–3649 (2022).
29. Kraus, A., Jainae, K., Unob, F. & Sukpirom, N. Synthesis of MPTS-modified cobalt ferrite

nanoparticles and their adsorption properties in relation to Au(III). *J. Colloid Interface Sci.* **338**, 359−365 (2009).

30. Yen, C.-H., Lien, H.-L., Chung, J.-S. & Yeh, H.-D. Adsorption of precious metals in water by dendrimer modifiedmagnetic nanoparticles. *J. Hazard. Mater.* **322**, 215−222 (2017).
31. Tang, J., Zhao, J., Wang, S., Zhang, L., Zhao, M., Huang, Z. & Hu, Y. Pre-modification strategy to prepare a novel Zr-based MOF for selective adsorption of Palladium(II) from solution. *Chem. Eng. J.* **407**, 127223 (2021).
32. Uheida, A., Iglesias, M., Fontàs, C. & Zhang, Y.; Muhammed, M. Adsorption behavior of platinum group metals (Pd, Pt, Rh) on nonylthiourea-coated Fe_3O_4 nanoparticles. *Sep. Sci. Technol.* **41**, 909−923 (2006).
33. Kang, T.; Park, Y.; Yi, J. Highly selective adsorption of Pt^{2+} and Pd^{2+} using thiol-functionalized mesoporous silica. *Ind. Eng. Chem. Res.* **43**, 1478−1484 (2004).
34. Wang, W., Li, X., Wang, R., Wang, J., Deng, H. & Tong, S. Three-dimensional Ti_3C_2 MXene-POSS/V_2O_3@C nanocomposite aerogel for ultrafast and selective recovery of gold (III) at low temperatures. *Chem. Eng. J.* **468**, 143453 (2023).
35. Rasoulzadeh, H., Sheikhmohammadi, A., Abtahi, M., Roshan, B. & Jokar, R. (2021). Eco-friendly rapid removal of palladium from aqueous solutions using alginate-diatomite magnano composite. *J. Environ. Chem. Eng.* **9**, 105954 (2021).
36. Zeng, C., Liu, P., Xiao, Z., Li, Y., Song, L., Cao, Z., Wu, D. & Zhang, Y.-F. Highly selective adsorption and recovery of palladium from spent catalyst wastewater by 1,4,7,10-tetraazacyclododecane-modified mesoporous silica. *ACS Sustainable Chem. Eng.* **10**, 1103−1114 (2022).
37. Sun, D. T., Gasilova, N., Yang, S., Oveisi, E. & Queen, W. L. Rapid, selective extraction of trace amounts of gold from complex water mixtures with a metal–organic framework (MOF)/polymer composite. *J. Am. Chem. Soc.* **140**, 16697−16703 (2018).
38. Lin, S., Reddy, D. H. K., Bediako, J. K., Song, M.-H., Wei, W., Kim, J.-A. & Yun, Y.-S. Effective

adsorption of Pd(II), Pt(IV) and Au(III) by Zr(IV)-based metal–organic frameworks from strongly acidic solutions. *J. Mater. Chem. A* **5**, 13557 (2017).

39. Wu, C., Zhu, X., Wang, Z., Yang, J., Li, Y. & Gu, J. Specific recovery and in situ reduction of precious metals from waste to create MOF composites with immobilized nanoclusters. *Ind. Eng. Chem. Res.* **56**, 13975–13982 (2017).
40. Lim, C.-R., Lin, S. & Yun, Y.-S. Highly efficient and acid-resistant metal-organic frameworks of MIL-101(Cr)-NH_2 for Pd(II) and Pt(IV) recovery from acidic solutions: Adsorption experiments, spectroscopic analyses, and theoretical computations. *J. Hazard. Mater.* **387**, 121689 (2020).
41. Guo, J., Fan, X., Wang, J., Yu, S., Laipan, M., Ren, X., Zhang, C., Zhang, L. & Li, Y. Highly efficient and selective recovery of Au(III) from aqueous solution by bisthiourea immobilized UiO-66-NH_2: Performance and mechanisms. *Chem. Eng. J.* **425**, 130588 (2021).
42. Tang, J., Chen, Y., Wang, S., Kong, D. & Zhang, L. Highly efficient metal-organic frameworks adsorbent for Pd(II) and Au(III) recovery from solutions: Experiment and mechanism. *Environ. Res.* **210**, 112870 (2022).
43. Chang, Z., Li, F., Qi, X., Jiang, B. & Kou, J. Selective and efficient adsorption of Au (III) in aqueous solution by Zr-based metal-organic frameworks (MOFs): An unconventional way for gold recycling. *J. Hazard. Mater.* **390**, 122175 (2020).
44. Liu, M., Long, X., Li, X., Du, Y., Zhao, Y., Huang, Z., Chen, Y. & Chen, R. Enrichment and activation of cyano in cobalt hexacyanoferrate for specific recovery of ultra-low concentrations of platinum. *Sep. Purif. Technol.* **328**, 124925 (2024).
45. Maponya, T. C., Makgopa, K., Somo, T. R., Tshwane, D. M. & Modibane, K. D. Highly adsorptive removal of palladium and platinum ions from wastewater using novel ethylenediamine-glutaraldehyde-grafted metal organic framework. *Environ. Nanotechnol. Monit. Manage.* **20**, 100805 (2023).
46. Nazri, S., Khajeh, M., Oveisi, A. R., Luque, R., Rodríguez-Castellón, E. & Ghaffari-Moghaddam, M. Thiol-functionalized PCN-222 MOF for fast and selective extraction of gold ions from aqueous media.

Sep. Purif. Technol. **259**, 118197 (2021).

47. Maponya, T. C., Modibane, K. D., Somo, T. R. & Makgopa, K. Selective adsorption of palladium ions from wastewater by ion-imprinted MIL-101 (Cr) derived from waste polyethylene terephthalate: Isotherms and kinetics. *Sep. Purif. Technol.* **307**, 122767 (2023).
48. Bratskaya, S., Privar, Y., Ustinov, A., Azarova, Y. & Pestov, A. Recovery of Au(III), Pt(IV), and Pd(II) using pyridylethyl-containing polymers: Chitosan derivatives vs synthetic polymers. *Ind. Eng. Chem. Res.* **55**, 10377−10385 (2016).
49. Mao, J., Lee, S. Y., Won, S. W. & Yun, Y.-S. Surface modified bacterial biosorbent with poly(allylamine hydrochloride): Development using response surface methodology and use for recovery of hexachloroplatinate (IV) from aqueous solution. *Water Res.* **44**, 5919−5928 (2010).
50. Kim, J., Kim, K. R., Hong, Y., Choi, S., Yavuz, C. T., Kim, J. W. & Nam, Y. S. Photochemically enhanced selective adsorption of gold ions on tannin-coated porous polymer Microspheres. *ACS Appl. Mater. Interfaces* **11**, 21915−21925 (2019).
51. Grad, O., Ciopec, M., Negrea, A., Duțeanu, N., Vlase, G., Negrea, P., Dumitrescu, C., Vlase, T. & Vodă, R. Precious metals recovery from aqueous solutions using a new adsorbent material. *Sci. Rep.* **11**, 2016 (2011).
52. Xue, D., Li, T., Liu, Y., Yang, Y., Zhang, Y., Cui, J. & Guo, D. Selective adsorption and recovery of precious metal ions from water and metallurgical slag by polymer brush graphene–polyurethane composite. *React. Funct. Polym.* **136**, 138−152 (2019).
53. Jung, Y., Do, T., Choi, U. S., Jung, K.-W. & Choi, J.-W. Cage-like amine-rich polymeric capsule with internal 3D center-radial channels for efficient and selective gold recovery. *Chem. Eng. J.* **438**, 135618 (2022).
54. Li, B., Xiong, W., Cao, Y., Zhou, X., Zhu, H., Li, M., Yang, L. & Shao, P. Targeting of platinum capture under 1+1 aqua regia using robust and recyclable polymeric polyamine resin: Adsorption performance and mechanism. *Environ. Res.* **227**, 115814 (2023).

55. Adhikari, C. R., Parajuli, D., Kawakita, H., Inoue, K., Ohto, K. & Harada, H. Dimethylamine-modified waste paper for the recovery of precious metals. *Environ. Sci. Technol.* **42**, 5486−5491 (2008).

56. Yu, D., Morisada, S., Kawakita, H., Sakaguchi, K., Osada, S., Ohto, K., Inoue, K., Song, S.-M., Zhang, G. & Sathuluri, R. R. Gold recovery from precious metals in acidic media by using human hair waste as a new pretreatment-free green material. *J. Environ. Chem. Eng.* **9**, 104724 (2021).

57. Gurung, M., Adhikari, B. B., Morisado, S., Kawakita, H., Ohto, K., Inoue, K. & Alam, S. *N*-aminoguanidine modified persimmon tannin: A new sustainable material for selective adsorption, preconcentration and recovery of precious metals from acidic chloride solution. *Bioresour. Technol.* **129**, 108−117 (2013).

58. Mincke, S., Asere, T. G., Verheye, I., Folens, K., Bussche, F. V., Lapeire, L., Verbeken, K., Voort, P. V. D., Tessema, D. A., Fufa, F., Laing, G. D. & Stevens, C. V. Functionalized chitosan adsorbents allow recovery of palladium and platinum from acidic aqueous solutions. *Green Chem.* **21**, 2295 (2019).

59. Wang, S., Vincent, T., Roux, J.-C., Faur, C. & Guibal, E. Pd(II) and Pt(IV) sorption using alginate and algal-based beads. *Chem. Eng. J.* **313**, 567−579 (2017).

60. Zhou, L., Liu, J. & Liu, Z. Adsorption of platinum(IV) and palladium(II) from aqueous solution by thiourea-modified chitosan microspheres. *J. Hazard. Mater.* **172**, 439−446 (2009).

61. Guo, J., Fan, X., Li, Y., Yu, S., Zhang, Y., Wang, L. & Ren, X. Mechanism of selective gold adsorption on ion-imprinted chitosan resin modified by thiourea. *J. Hazard. Mater.* **415**, 125617 (2021).

62. Liu, L., Liu, S., Zhang, Q., Li, C., Bao, C., Liu, X. & Xiao, P. Adsorption of Au(III), Pd(II), and Pt(IV) from aqueous solution onto graphene oxide. *J. Chem. Eng. Data* **58**, 209−216 (2013).

63. Zalupski, P. R., McDowell, R. & Dutech, G. The adsorption of gold, palladium, and platinum from acidic chloride solutions on mesoporous carbons. *Solvent Extr. Ion Exch.* **32**, 737−748 (2014).

64. Sharma, S. & Rajesh, N. Synergistic influence of graphene oxide and tetraoctylammonium bromide

(frozen ionic liquid) for the enhanced adsorption and recovery of palladium from an industrial catalyst. *J. Environ. Chem. Eng.* **4**, 4287–4298 (2016).

65. Wang, Z., Xu, X., Ma, S., Wang, H., Zhao, H., Wang, Y., Tong, S., Su, Z., Wang, W. & Bai, J. The superior adsorption capacity of boron-nitrogen co-doping walnut shell biochar powder for Au(III), Pt(IV), and Pd(II). *J. Environ. Chem. Eng.* **9**, 106288 (2021).
66. Saha, S., Venkatesh, M., Basu, H., Pimple, M. V. & Singhal, R. K. Recovery of gold using graphene oxide/calcium alginate hydrogel beads from a scrap solid state detector. *J. Environ. Chem. Eng.* **7**, 103134 (2019).
67. Ianăşi, C., Svera, P., Popa, A., Lazău, R., Negrea, A., Negrea, P., Duteanu, N., Ciopec, M. & Nemes, N.-S. Adsorbent material based on carbon black and bismuth with tunable properties for gold recovery. *Materials* **16**, 2837 (2023).
68. Pei, X., Qu, Y., Shen, W., Li, H., Xhang, X., Li, S., Zhang, Z. & Li, X. Green synthesis of gold nanoparticles using fungus *Mariannaea* sp. HJ and their catalysis in reduction of 4-nitrophenol. *Environ. Sci. Pollut. Res.* **24**, 21649–21659 (2017).
69. Majumdar, R., Bag, B. G. & Ghosh, P. *Mimusops elengi* bark extract mediated green synthesis of gold nanoparticles and study of its catalytic activity. *Appl. Nanosci.* **6**, 521–528 (2016).
70. Wu, S., Yan, S., Huang, R., Cui, J., Su, R. & He, Z. Green synthesis of gold nanoparticles using aspartame and their catalytic activity for *p*-nitrophenol reduction. *Nanoscale Res. Lett.* **10**, 213 (2015).
71. Khan, S., Runguo, W., Tahir, K., Jichuan, Z. & Zhang, L. Catalytic reduction of 4-nitrophenol and photo inhibition of *Pseudomonas aeruginosa* using gold nanoparticles as photocatalyst. *J. Photoch. Photobio. B* **170**, 181–187 (2017).
72. Gao, Z., Su, R., Huang, R., Qi, W. & He, Z. Glucomannan-mediated facile synthesis of gold nanoparticles for catalytic reduction of 4-nitrophenol. *Nanoscale Res. Lett.* **9**, 404 (2014).
73. Bordbar, M. & Mortazavimanesh, N. Green synthesis of Pd/walnut shell nanocomposite using *Equisetum arvense* L. leaf extract and its application for the reduction of 4-nitrophenol and organic

dyes in a very short time. *Environ. Sci. Pollut. Res.* **24**, 4093–4104 (2017).

74. Sargin, I., Barab, T. & Arslan, G. Environmental remediation by chitosan-carbon nanotube supported palladium nanoparticles: Conversion of toxic nitroarenes into aromatic amines, degradation of dye pollutants and green synthesis of biaryls. *Sep. Purif. Technol.* **247**, 116987 (2020).
75. Baran, N. Y., Baran, T., Nasrollahzadeh, M. & Varma, R. S. Pd nanoparticles stabilized on the Schiff base-modified boehmite: Catalytic role in Suzuki coupling reaction and reduction of nitroarenes. *J. Organomet. Chem.* **900**, 120916 (2019).
76. Pandey, S. & Mishra, S. B. Catalytic reduction of *p*-nitrophenol by using platinum nanoparticlesstabilised by guar gum. *Carbohydr. Polym.* **113**, 525–531 (2014).
77. Ullah, S., Ahmad, A., Wang, A., Raza, M., Jan, A. U., Tahir, K., Rahman, A. U. & Qipeng, Y. Bio-fabrication of catalytic platinum nanoparticles and their in vitro efficacy against lungs cancer cells line (A549). *J. Photoch. Photobio. B* **173**, 368–375 (2017).
78. Umamaheswari, C., Lakshmanan, A. & Nagarajan, N. S. Green synthesis, characterization and catalytic degradation studies of gold nanoparticles against Congo Red and Methyl Orange. *J. Photoch. Photobio. B* **178**, 33–39 (2018).
79. Iqbal, K., Iqbal, A., Kirilov, A. M., Wang, B., Liu, W. & Tang, Y. A new Ce-doped MgAl-LDH@Au nanocatalyst for highly efficient reductive degradation of organic contaminants. *J. Mater. Chem. A* **5**, 6716 (2017).
80. Shah, S. A., Ahmad, Z., Khan, S. A., Al-Ghamdi, Y. O. Bakhsh, E. M., Khan, N., Rehman, M. u., Jabli, M. & Khan, S. B., Biomass impregnated zero-valent Ag and Cu supported-catalyst: Evaluation in the reduction of nitrophenol and discoloration of dyes in aqueous medium. *J. Organomet. Chem.* **938**, 121756 (2021).
81. Alla, S. K., Verma, A. D., Kumar, V., Mandal, R. K., Sinha I. & Parsad, N. K., Solvothermal synthesis of CuO–MgO nanocomposite particles and their catalytic applications. *RSC Adv.* **6**, 61927 (2016).
82. Iqbal, K., Iqbal, A., Kirilov, A. M., Liu, W. & Tang, Y. Hybrid metal–organic-framework/inorganic

nanocatalyst toward highly efficient discoloration of organic dyes in aqueous medium. *Inorg. Chem.* **57**, 13270−13278 (2018).

83. Malik, A. & Nath, M. Ultrafast catalytic reduction of toxic nitroaromatics and organic colouring dyes by using Au/ZIF-11: Efficient wastewater treatment. *J. Water Process Eng.* **44**, 102362 (2021).
84. Sharma, S. & Rajesh, N. Augmenting the adsorption of palladium from spent catalyst using a thiazole ligand tethered on an amine functionalized polymeric resin. *Chem. Eng. J.* **283**, 999−1008 (2016).
